# Performance of large language models in the differential diagnosis of benign and malignant biliary stricture

**DOI:** 10.3389/fonc.2025.1613818

**Published:** 2025-07-03

**Authors:** Chenxi Kang, Jing Li, Xintian Yang, Gui Ren, Linhui Zhang, Wei Wang, Xin Liu, Lei Wang, Guochen Shang, Jianglong Hong, Bingnian Wan, Yu Du, Wei Zeng, Yaling Liu, Tongxin Li, Lijun Lou, Hui Luo, Shuhui Liang, Yong Lv, Yanglin Pan

**Affiliations:** ^1^ Xijing Hospital of Digestive Diseases, Air Force Medical University, Xi’an, China; ^2^ Department of Gastroenterology, People’s Liberation Army Joint Logistics Support Force 940th Hospital, Lanzhou, Gansu, China; ^3^ Department of Gastroenterology, Third People’s Hospital of Gansu Province, Lanzhou, Gansu, China; ^4^ Department of Gastroenterology, Ankang Traditional Chinese Medicine Hospital, Ankang, China; ^5^ Tongji Medical College, Huazhong University of Science and Technology, Wuhan, Hubei, China; ^6^ First Affiliated Hospital of Anhui Medical University, Hefei, Anhui, China; ^7^ Yantai Ludong Hospital, Shandong Provincial Hospital Group, Yantai, Shandong, China; ^8^ Department of Gastroenterology, Qinzhou Second People’s Hospital, Qinzhou, China; ^9^ Xiang’an Hospital, Xiamen University, Xiamen, Fujian, China

**Keywords:** large language model, biliary stricture, cholangiocarcinoma, prediction model, diagnosis

## Abstract

**Background:**

Distinguishing benign from malignant biliary strictures remains challenging. Large Language Models (LLMs) show promise in enhancing diagnostic accuracy. This study aimed to evaluate the performances of ten LLMs in the differential diagnosis of benign and malignant biliary strictures.

**Methods:**

Consecutive patients with biliary strictures undergoing endoscopic retrograde cholangiopancreatography (ERCP) at Xijing Hospital between January and December 2024 were retrospectively analyzed. Ten LLMs were systematically prompted with standardized clinical, laboratory, and imaging data. Performance was compared against tumor markers (CA19-9, CEA), a new multivariable clinical model, and ten independent pancreaticobiliary exoerienced physicians. Subgroup analyses assessed hilar (n=29) versus non-hilar strictures. Gold-standard diagnosis relied on histopathology and ≥3-month follow-up.

**Results:**

Among the 159 included patients (83 benign, 76 malignant), four LLMs (Kimi, Deepseek-R1, Claude-3.5S, Llama-3.1), the clinical model (AUC:0.83), and six physicians achieved >80% accuracy. Kimi demonstrated superior accuracy (87%), significantly outperforming 70% of physicians (7/10, p<0.01). Three other LLMs (Deepseek-R1:83%, Claude-3.5S:82%, Llama-3.1:81%) and the clinical model performed comparably to physicians (78-84%, p>0.05), collectively surpassing tumor markers (CA19–9 accuracy:66%, CEA:71%). Physicians demonstrated higher accuracy for hilar strictures (87% vs. 79% for non-hilar, p<0.001). LLMs showed similar performance across stricture locations (hilar:64-95%; non-hilar:62-88%, p>0.05). For hilar strictures, 7/10 physicians achieved significantly higher accuracy (87-90%) than 8/10 LLMs (64-84%, p<0.05).

**Conclusions:**

Using clinical, lab, and imaging data, some LLMs achieved diagnostic accuracy comparable to or exceeding clinical models and experienced physicians for differentiating benign versus malignant strictures. However, for hilar strictures, LLM performance was inferior to over half of the physicians.

## Introduction

Biliary strictures, characterized by abnormal bile duct narrowing, can significantly obstruct bile flow. An estimated 54-87% of biliary strictures are malignant (1–5), arising from local or metastatic cancers. Benign biliary strictures have heterogeneous etiologies including surgical bile duct injury, chronic pancreatitis, or chronic cholangiopathies (e.g., primary sclerosing cholangitis) (6). Benign and malignant strictures differ significantly in management and prognosis. Benign strictures are typically managed by endoscopic dilation, stenting, or surgery (7–9), while malignant strictures require aggressive approaches including surgical resection, palliative drainage, and systemic therapy (10–12). Thus, accurately distinguishing between them is crucial for guiding treatment and prognostic assessment.

Characteristics of biliary strictures are typically assessed via brushing cytology, forceps biopsy, or cholangioscopic biopsy during endoscopic retrograde cholangiopancreatography (ERCP) (13). Brush cytology and forceps biopsy demonstrate diagnostic accuracies of 15–80% (14, 15), while cholangioscopic biopsy achieves 70–87% (13, 16). Endoscopic ultrasound-guided (EUS) fine needle aspiration (FNA) or fine needle biopsy (FNB) shows favorable accuracy, particularly for extrinsic mass-related strictures (1). Advanced techniques like intraductal ultrasonography (IDUS) (17), probe-based confocal laser endomicroscopy (pCLE) (18), and optical coherence tomography (OCT) (19), offer enhanced precision but are limited by invasiveness, cost, and availability.

Significant differences also exist in common clinical parameters between benign and malignant strictures, including age of onset, duration of liver function abnormalities, previous surgeries, and tumor markers. These noninvasive, accessible parameters facilitate convenient prediction. Wang et al. used CA50, CA19-9, and AFP to achieve an AUC of 0.879 (95% CI: 0.841–0.917) (20), while Zhang et al. combined MRI with inflammatory markers for an AUC of 0.802 (95% CI: 0.719–0.870) (18). Though promising, these models require further validation.

LLMs including Deepseek-R1, GPT-4T, Claude-3.5S, and Llama-3.1 show potential in improving diagnostic accuracy across medicine (21–24). Trained on vast medical data, LLMs may assist preliminary diagnosis by reducing interpretational variability. However, their utility for differentiating biliary strictures—a specialized, less common condition—remains unknown. We hypothesize that LLMs leveraging common clinical data (manifestations, blood tests, imaging) could aid this differentiation.

In this study, we aimed to evaluate the diagnostic performance of ten distinct LLMs for diagnosing benign versus malignant biliary strictures, comparing performance against tumor markers, a novel clinical model, and ten experienced pancreaticobiliary specialists.

## Methods

### Study design

This retrospective study evaluated the diagnostic performance of ten distinct large language models (LLMs) in differentiating between benign and malignant biliary strictures. The study protocol was approved by the Ethics Committee of Xijing Hospital. The written informed consent was obtained from all the patients or their next of kin.

### Patients

Consecutive patients aged ≥18 years admitted to Xijing Hospital for biliary stricture evaluation between January and December 2024 were eligible. Inclusion criteria required a definitive etiological diagnosis confirmed by pathological examination and regular follow-up exceeding 3 months. Patients with incomplete medical records were excluded. Malignancy diagnosis relied on pathological results obtained via endoscopic retrograde cholangiopancreatography (ERCP), percutaneous transhepatic cholangiographic drainage (PTCD), endoscopic ultrasound (EUS), biopsy, or surgery. Benign strictures required confirmation by benign pathology and absence of progression over ≥3 months.

### Data collection

**Figure 1:**
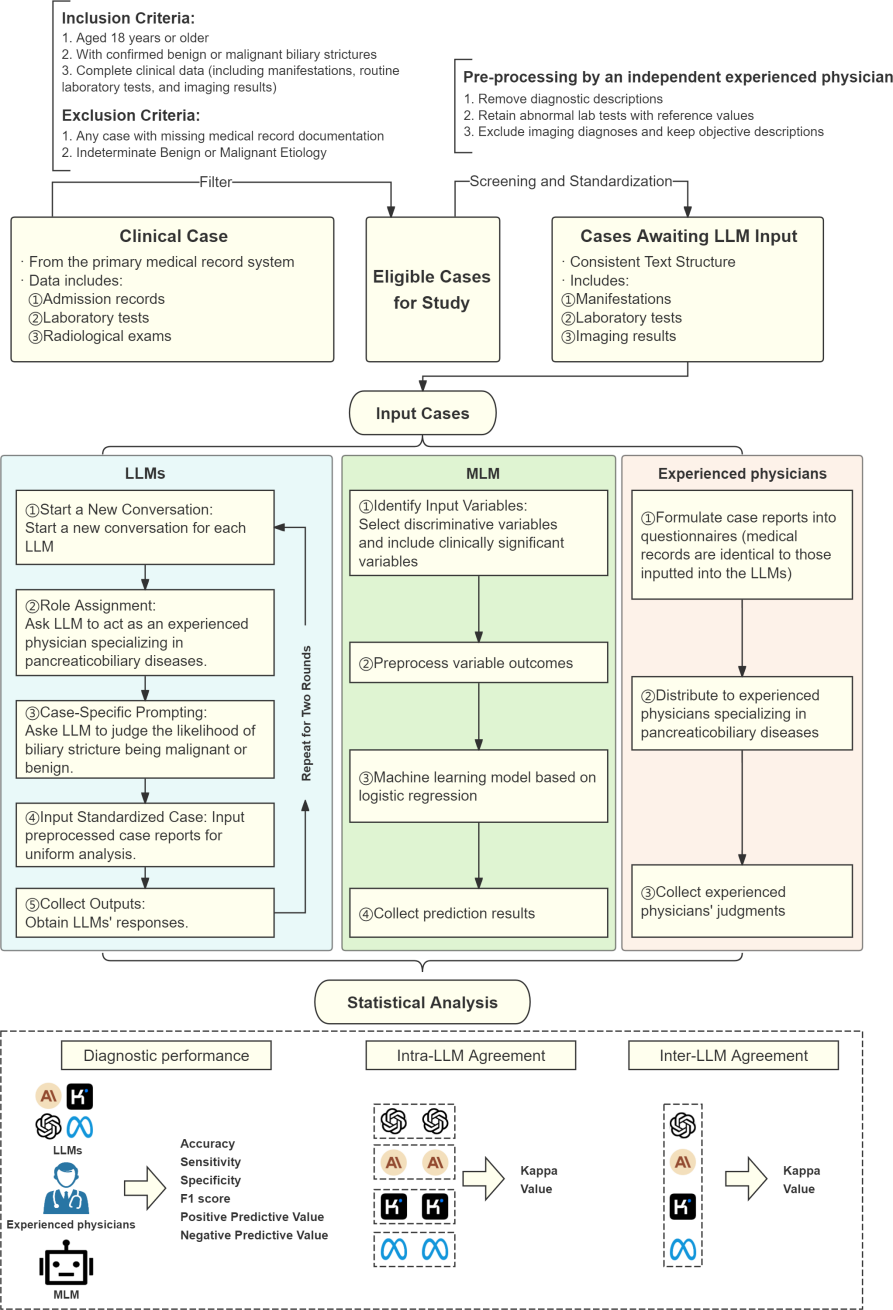
Flowchart of overall study design. LLM, Large Language Model; MLM, Machine Learning Model.

Demographic, clinical, imaging, and pathological data were extracted from electronic medical records. An independent physician standardized data inputs for ten LLMs, ensuring unbiased differentiation. Case data was structured uniformly, excluding diagnostic conclusions, and included clinical presentation, history, imaging reports (CT, MRI, MRCP), and lab results (complete blood count, liver and renal function tests, lipids, coagulation, tumor/inflammatory markers). Any indications of benignancy or malignancy from the reports were removed. A flowchart of the overall study design is shown in **Figure 1**, illustrating the process from patient selection to diagnostic performance evaluation.

### Large language models for differential diagnosis of biliary strictures

Ten LLMs were selected for evaluation for reproducibility, including 1) mainstream commercial models with proven medical reasoning capabilities in prior studies (GPT-4T, GPT-4o, Gemini-1.5 pro, Claude-3.5S), 2) models developed by Chinese companies to align with the Chinese-language clinical data (Kimi, ERNIE-4, Qwen-2, GLM-4); 3) open-source models (Deepseek-R1, Llama-3.1) (25, 26). To ensure consistency and reproducibility, a structured query approach was used. A case-specific prompt simulated consultation with an experienced pancreaticobiliary specialist: “Supposing you are an experienced physician specializing in pancreaticobiliary diseases, when encountering a case with biliary stricture, please provide a tentative judgment indicating whether the cause of the stricture is more likely to be malignant or benign.” (Detailed prompt provided in **Supplementary Method S1**). For each LLM, a new conversation session was initiated to eliminate contextual carryover. Probabilistic LLM outputs (e.g., “likely benign,” “possibly malignant”) were converted into binary outcomes (benign/malignant) using predefined rules. Responses indicating malignancy (e.g., “likely malignant,” “probably malignant,” “suggestive of malignancy”) were classified as “malignant.” Responses indicating benignancy (e.g., “likely benign,” “probably benign,” “suggestive of a benign process”) were classified as “benign.” Explicit statements (“benign”/”malignant”) were directly categorized.

All queries employed identical deterministic parameters: temperature=0.0 (output consistency), max tokens=10240, and consistent system prompts. Queries were executed in January 2025 using contemporaneous model versions. The evaluated LLMs included: Deepseek-R1, GPT-4T, GPT-4o, Claude-3.5S, Gemini-1.5 Pro, Kimi, Llama-3.1 405B, ERNIE-4.0-Turbo-8K, Qwen-2-72B, and ChatGLM-4-9B (details in **Supplementary Table S1**). Chain-of-thought (CoT) outputs were extracted for reasoning pattern analysis. Cases were categorized by diagnostic accuracy (correct/incorrect), and reasoning traces were independently assessed by two gastroenterologists using predefined clinical logic criteria (**Supplementary Table S3**).

### Development of a clinical prediction model

A multivariable logistic regression model incorporating key demographic, clinical, laboratory, and imaging parameters was developed. The modeling process initiated with univariable screening to identify candidate variables (p < 0.10), followed by multivariable regression analysis. Variables were retained multivariable models based on statistical significance (p < 0.05) or established clinical relevance for variables approaching significance. Collinearity was assessed using variance inflation factors (VIF) and Spearman correlation coefficients (|ρ| > 0.6), with clinical importance determining variable selection when collinearity was detected. Bidirectional stepwise selection (forward/backward) using Akaike (AIC) and Bayesian (BIC) information criteria optimized the model. Final model selection prioritized minimized AIC/BIC and maximized predictive performance, with internal validation implemented through bootstrapping (1,000 resamples). CA19–9 and CEA were separately assessed as independent diagnostic markers.

### Experienced physicians’ evaluation

Ten experienced pancreaticobiliary specialists (each with ≥10 years of clinical practice) independently evaluated comprehensive clinical summaries identical to those processed by the LLMs. All diagnostic predictions regarding benign or malignant status were made without intercommunication among physicians to ensure independent assessment.

### Outcome

The primary outcome was diagnostic accuracy that was calculated as the average of sensitivity and specificity to account for class imbalance (range: 0-1, higher values superior). For LLMs, the highest accuracy from duplicate assessments was selected. Secondary performance metrics included sensitivity (proportion of true positives correctly identified), specificity (proportion of true negatives correctly identified), positive predictive value (PPV, proportion of positive predictions that were correct), negative predictive value (NPV, proportion of negative predictions that were correct), and F1-score (harmonic mean of precision and sensitivity providing balanced assessment).

### Statistical analysis

Continuous variables are presented as mean ± standard deviation (normally distributed) or median [interquartile range] (non-normal distributions), while categorical variables are expressed as proportions (%) with odds ratios (OR) and 95% confidence intervals (CI) for association analyses. McNemar and DeLong tests were used to compare performance metrics between models. Confidence intervals for accuracy, sensitivity, and specificity were calculated using the Clopper-Pearson method, with proportion differences reported in pairwise comparisons. Subgroup analysis evaluated LLM diagnostic performance by stricture location (hilar vs. non-hilar). Internal concordance of LLMs was assessed through weighted Cohen’s kappa (κ) with 95% CI based on duplicate assessments, interpreted as: κ ≤ 0.20 (slight concordance), 0.21-0.40 (fair), 0.41-0.60 (moderate), 0.61-0.80 (substantial), and 0.81-1.00 (almost perfect). Pairwise comparisons of classification accuracy employed McNemar’s test with Holm-Bonferroni correction for multiple comparisons (family-wise α = 0.05; significance threshold: adjusted p < 0.05). All statistical tests were two-sided. Statistical analyses were conducted using R version 4.3.1.

## Results

### Patient demographics and clinical characteristics

During the study period, 270 patients were diagnosed with biliary stricture, of whom 111 were excluded according to inclusion/exclusion criteria, resulting in a final cohort of 159 patients (83 benign, 76 malignant). Baseline demographic and clinical characteristics are summarized in **Table 1**. Compared with benign stricture patients, those with malignant lesions were older (65.13 vs. 57.19 years), exhibited higher bilirubin (149.14 vs. 78.15 μmol/L, p < 0.001) and CA19–9 levels (2888.33 vs. 246.32 U/mL, p = 0.003), a higher proportion of lymph node enlargement (47.4% vs. 19.3%, p < 0.001), and more frequent hilar strictures (26.3% vs. 10.8%, p = 0.020).

**Table 1:** Patient demographics, clinical characteristics, and diagnostic markers.

Variables	Overall (n=159)	Benign (n=83)	Malignant (n=76)	P value
Age (year)	60.99 (14.03)	57.19 (16.22)	65.13 (9.70)	<0.001
Male, n (%)	66 (41.5)	35 (42.2)	31 (40.8)	0.988
BMI (kg/m^2^)	20.91 (3.43)	21.55 (3.20)	20.17 (3.58)	0.075
Prior surgical history *, n (%)	85 (53.5)	51 (61.4)	34 (44.7)	0.051
Disease duration < 1month †, n (%)	135 (61.4)	69 (57.0)	66 (66.7)	0.005
Traditional tumor markers				
CA125 (U/ml)	48.36 (131.48)	26.23 (51.27)	72.53 (180.03)	0.026
CA19-9 (U/ml)	1435.23 (6681.62)	246.32 (1854.95)	2888.33 (9574.67)	0.003
CEA (ng/ml)	15.00 (77.00)	3.22 (3.21)	29.40 (113.39)	0.012
AFP (ng/ml)	5.72 (17.88)	5.04 (15.03)	6.47 (20.62)	0.616
Whole blood tests				
White blood cell (cell/μL)	6570 (3050)	6410 (2420)	6740 (3620)	0.491
Hemoglobin (g/dL)	12.20 (2.05)	12.75 (1.90)	11.67 (2.06)	0.002
Platelet count (× 103/μL)	214.06 (79.79)	206.84 (76.57)	221.94 (82.95)	0.235
Liver/renal function tests				
Total bilirubin (μmol/L)	112.52 (119.36)	78.15 (105.85)	149.14 (122.78)	<0.001
Albumin (g/dL)	3.78 (0.68)	3.94 (0.66)	3.61 (0.66)	0.002
Creatinine (mg/dL)	0.85 (0.18)	0.84 (0.18)	0.85 (0.17)	0.668
Sodium (mEq/L)	141.96 (2.83)	141.92 (2.84)	142.00 (2.84)	0.858
Coagulation profiles				
International normalized ratio	1.09 (0.16)	1.10 (0.18)	1.09 (0.14)	0.605
Lipid profiles				
Total cholesterol (mg/dL)	169.50 (41.70)	171.81 (44.79)	167.18 (37.84)	0.477
Triglyceride (mg/dL)	111.50 (86.73)	110.62 (73.45)	112.39 (100.00)	0.859
Inflammatory markers				
CRP (mg/dL)	2.02 (3.18)	2.21 (3.48)	1.81 (2.83)	0.431
IL6 (pg/ml)	33.43 (363.27)	3.20 (2.72)	66.45 (525.24)	0.274
PCT (ng/ml)	1.14 (4.06)	0.60 (1.79)	1.73 (5.53)	0.08
Immune abnormalities‡, n (%)	26 (16.4)	11 (13.3)	15 (19.7)	0.174
Lymph node enlargement, n (%)	52 (32.7)	16 (19.3)	36 (47.4)	<0.001
Biliary stricture sites, n (%)				0.020
Hilar	29 (18.2)	9 (10.8)	20 (26.3)	
Non-hilar	130 (81.8)	74 (89.2)	56 (73.7)	

Data are mean (standard deviation) or numbers (percentages) unless otherwise specified. BMI, Body Mass Index; CA19-9, Carbohydrate Antigen 199; CEA, Carcinoembryonic Antigen; AFP, Alpha-Fetoprotein; CA125, Carbohydrate Antigen 125; CRP, C-Reactive Protein; IL6, Interleukin6; PCT, Procalcitonin.

*Surgical history was defined as a history of liver transplantation and biliary surgery.

†Disease duration < 1 month was defined as a period less than one month from the onset of symptoms.

‡Immune abnormalities were defined as the presence of IgG4 subclass abnormalities, autoimmune diseases, or abnormalities detected in a series of autoantibody tests.

### Clinical data-driven diagnostic model performance

**Figure 2:**
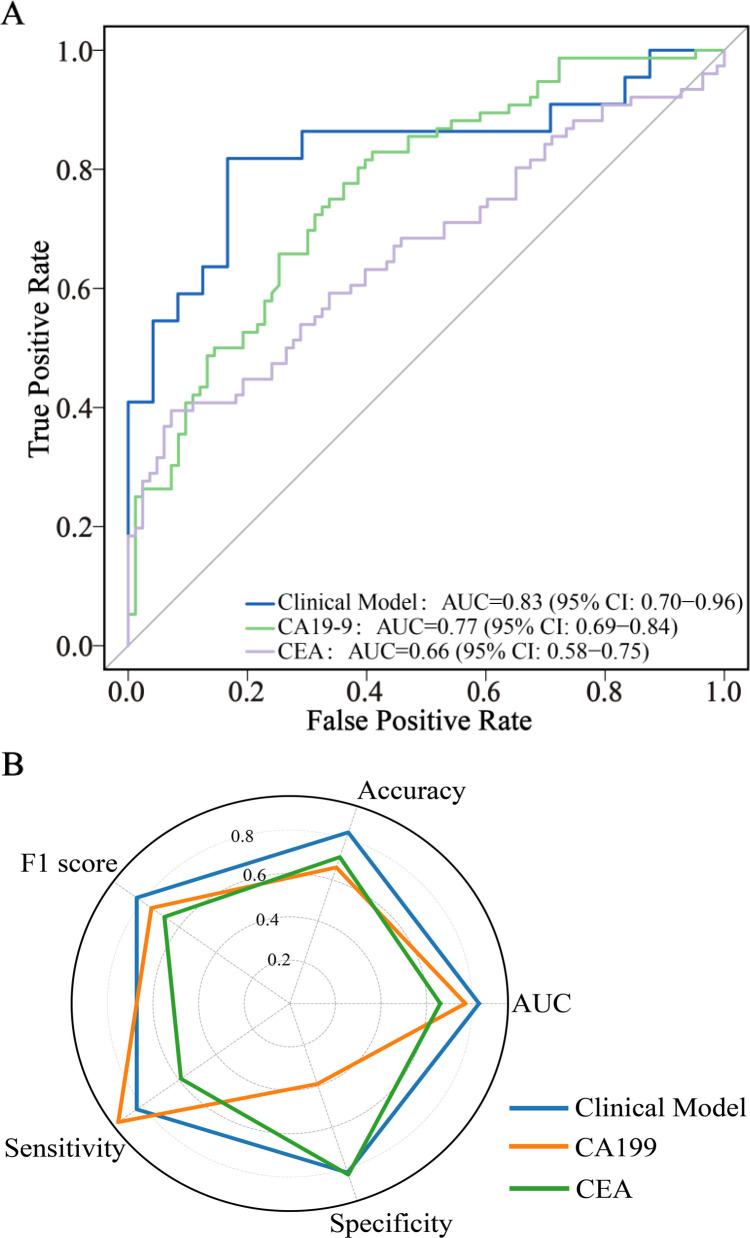
ROC curve analysis **(A)** and radar chart **(B)** of diagnostic model and tumor markers. CA19-9, Carbohydrate Antigen 199; CEA, Carcinoembryonic Antigen; AUC, Area Under the Curve; CI, Confidence Interval; ROC, Receiver Operating Characteristic.

A stepwise multivariable logistic regression was employed to develop the diagnostic model, with the final model retaining age, CA19-9, CEA, disease duration <1 month, C-reactive protein (CRP), surgical history, and lymph node enlargement (**Table 2**). **Supplementary Figure S1** illustrates the impact of hyperparameter λ on model accuracy under L2 regularization, with the optimal λ value of 149.3 determined via 10-fold cross-validation to maximize test-set accuracy. The model achieved an AUC of 0.83 (95% CI: 0.70–0.96), accuracy of 0.83, sensitivity of 0.83, and specificity of 0.82 (**Table 3**, **Figure 2**), outperforming tumor markers alone. CA19–9 showed an AUC of 0.77 (95% CI: 0.69–0.84), accuracy of 0.66, specificity of 0.93, and sensitivity of 0.39; CEA yielded an AUC of 0.66 (95% CI: 0.58–0.75), accuracy of 0.71, specificity of 0.59, and sensitivity of 0.83. Internal validation via bootstrapping (1000 iterations) confirmed consistent AUC of 0.83 (**Supplementary Figure S2**).

**Table 2:** Selection of variables based on univariate and multivariate logistic regression analysis.

Variables	Univariate Logistic Regression		Multivariate Logistic Regression	
	OR (95%CI)	P	OR (95%CI)	P
Age > 55 (year)	4.53 (2.09, 9.8)	<0.001	4.68 (1.65, 14.51) §	0.005
Male	1.06 (0.56, 1.99)	0.86		
Prior Surgical history *	0.51 (0.27, 0.96)	0.04	0.74 (0.27, 1.99) §1	0.549
Disease duration < 1mouth †	2.79 (1.4, 5.57)	<0.001	3.48 (1.32, 9.79) §	0.014
Traditional tumor markers				
CA125 > 20 (U/ml)	3.79 (1.95, 7.35)	<0.001	1.74 (0.64, 4.81)	0.278
CA19-9 > 30(U/ml)	6.98 (3.33, 14.64)	<0.001	5.20 (1.81, 16.16) §	0.003
CEA > 5 (ng/ml)	8.37 (3.24, 21.63)	<0.001	4.99 (1.58, 18.14) §	0.009
AFP > 4 (ng/ml)	0.44 (0.23, 0.87)	0.02	1.32 (0.48, 3.73)	0.596
Whole blood tests				
White blood cell > 6000 (cell/μL)	0.52 (0.28, 0.99)	0.05	0.53 (0.21, 1.32)	0.179
Hemoglobin > 12.5 (g/dL)	0.50 (0.26, 0.96)	0.04	0.71 (0.30, 2.23)	0.487
Platelet count > 250 (× 103/μL)	1.51 (0.79, 2.90)	0.21		
Liver/renal function tests				
Total bilirubin > 2 (mg/dL)	5.14 (2.55, 10.39)	<0.001		
Albumin > 4 (g/dL)	0.38 (0.20, 0.75)	<0.001	0.53 (0.18, 1.49)	0.23
Creatinine > 0.75 (mg/dL)	1.67 (0.82, 3.40)	0.15		
Sodium > 140 (mEq/L)	1.51 (0.80, 2.85)	0.21		
Coagulation profiles				
International normalized ratio > 1	2.04 (0.94, 4.47)	0.07		
Inflammatory markers				
CRP > 0.6 (mg/dL)	0.42 (0.21, 0.82)	0.01	0.23 (0.08, 0.63)	0.006
IL6 > 1 (pg/ml)	2.52 (1.07, 5.92)	0.03	2.72 (0.81, 9.81)	0.112
PCT > 0.1 (ng/ml)	1.77 (0.94, 3.33)	0.08		
Immune abnormal ‡	1.61 (0.69, 3.76)	0.27		
Lymph node enlargement	3.77 (1.86, 7.64)	<0.001	2.90 (0.97, 9.16) §	0.06
Biliary stricture sites: Hilar	0.34 (0.14, 0.80)	0.01	0.50 (0.13, 1.77)	0.29

CA19-9, Carbohydrate Antigen 199; CEA, Carcinoembryonic Antigen; AFP, Alpha-Fetoprotein; CA125, Carbohydrate Antigen 125; CRP, C-Reactive Protein; IL6, Interleukin6; PCT, Procalcitonin; OR, odds ratio; CI, Confidence Interval; NA, Not Applicable.

*Surgical history was defined as a history of liver transplantation and biliary tract surgery.

†Disease duration was defined as a period less than one month from the onset of symptoms.

‡Immune abnormal was defined as the presence of immunoglobulin subclass 4 abnormalities, or having an autoimmune disease, or abnormalities in a series of autoantibody tests.

§Variables included in the clinical model.

§1Variables included based on clinical relevance despite borderline univariate p-values (p<0.05).

**Table 3:** Performance metrics of LLMs, EPs, clinical model and tumor markers.

Predictors	Acc, (95%CI)	Sens	Spec	F1	PPV	NPV	AUC, (95%CI)
LLMs							
GPT-4T	0.79 (0.71, 0.84)	0.59 (0.51, 0.64)	0.99 (0.92, 1.00)	0.74	0.98	0.69	–
GPT-4o	0.66 (0.57, 0.72)	0.34 (0.30, 0.39)	0.99 (0.92, 1.00)	0.50	0.97	0.58	–
Claude-3.5S	0.82 (0.74, 0.87)	0.71 (0.65, 0.76)	0.92 (0.88, 0.97)	0.80	0.91	0.74	–
Gemini-1.5 pro	0.62 (0.52, 0.68)	0.25 (0.19, 0.30)	0.99 (0.92, 1.00)	0.40	0.95	0.55	–
Kimi	0.87 (0.81, 0.92)	0.83 (0.77, 0.89)	0.91 (0.85, 0.96)	0.87	0.91	0.83	–
ERNIE-4	0.79 (0.71, 0.85)	0.66 (0.61, 0.72)	0.92 (0.88, 0.95)	0.76	0.90	0.71	–
Llama-3.1	0.81 (0.73, 0.86)	0.65 (0.60, 0.72)	0.97 (0.91, 0.99)	0.78	0.96	0.72	–
Qwen-2	0.76 (0.68, 0.82)	0.65 (0.60, 0.71)	0.87 (0.82, 0.92)	0.73	0.84	0.69	–
GLM-4	0.77 (0.69, 0.83)	0.69 (0.62, 0.75)	0.86 (0.82, 0.90)	0.75	0.84	0.71	–
Deepseek-R1	0.83 (0.76, 0.89)	0.81 (0.77, 0.86)	0.86 (0.81, 0.91)	0.83	0.86	0.80	–
Experienced Physicians							
EP1	0.84 (0.78, 0.90)	0.87 (0.82, 0.91)	0.82 (0.77, 0.88)	0.85	0.84	0.85	–
EP2	0.80 (0.73, 0.86)	0.80 (0.73, 0.85)	0.80 (0.75, 0.86)	0.80	0.81	0.78	–
EP3	0.79 (0.71, 0.84)	0.64 (0.59, 0.71)	0.93 (0.85, 0.96)	0.75	0.91	0.70	–
EP4	0.81 (0.74, 0.87)	0.84 (0.79, 0.88)	0.78 (0.72, 0.85)	0.82	0.80	0.82	–
EP5	0.81 (0.74, 0.87)	0.81 (0.77, 0.87)	0.82 (0.76, 0.88)	0.82	0.83	0.79	–
EP6	0.80 (0.73, 0.86)	0.76 (0.70, 0.82)	0.84 (0.79, 0.88)	0.80	0.84	0.76	–
EP7	0.79 (0.71, 0.85)	0.70 (0.65, 0.76)	0.88 (0.82, 0.93)	0.77	0.87	0.73	–
EP8	0.80 (0.72, 0.85)	0.66 (0.61, 0.72)	0.93 (0.88, 0.96)	0.77	0.92	0.72	–
EP9	0.78 (0.70, 0.84)	0.71 (0.66, 0.78)	0.84 (0.79, 0.89)	0.77	0.83	0.73	–
EP10	0.79 (0.72, 0.85)	0.77 (0.71, 0.83)	0.82 (0.78, 0.89)	0.80	0.82	0.77	–
Clinical Model							
Clinical Model	0.83 (0.69, 0.92)	0.83 (0.77, 0.88)	0.82 (0.76, 0.89)	0.83	0.83	0.82	0.83, (0.70, 0.96)
Tumor markers							
CA19-9	0.66 (0.59, 0.75)	0.93 (0.88, 0.97)	0.39 (0.35, 0.44)	0.75	0.63	0.83	0.77, (0.69, 0.84)
CEA	0.71 (0.63, 0.77)	0.59 (0.55, 0.64)	0.83 (0.78, 0.59)	0.68	0.79	0.65	0.66, (0.58, 0.75)
P *	0.016	0.201	0.769	0.678	1.000	0.769	0.387
P †	<0.001	0.013	0.646	0.029	0.009	0.646	0.031

CA19-9, Carbohydrate Antigen 199; CEA, Carcinoembryonic Antigen; AUC, Area Under the Curve; Acc, Accuracy; Sens, Sensitivity; Spec, Specificity; F1, F1 score; PPV, Positive Predictive Value; NPV, Negative Predictive Value; CI, Confidence Interval; EP, Experienced Physician.

*p value of Logistic Prediction Model versus CA19-9.

†p value of Logistic Prediction Model versus CEA.

### Comparative diagnostic performance of LLMs, tumor markers, clinical model, and physicians

**Figure 3:**
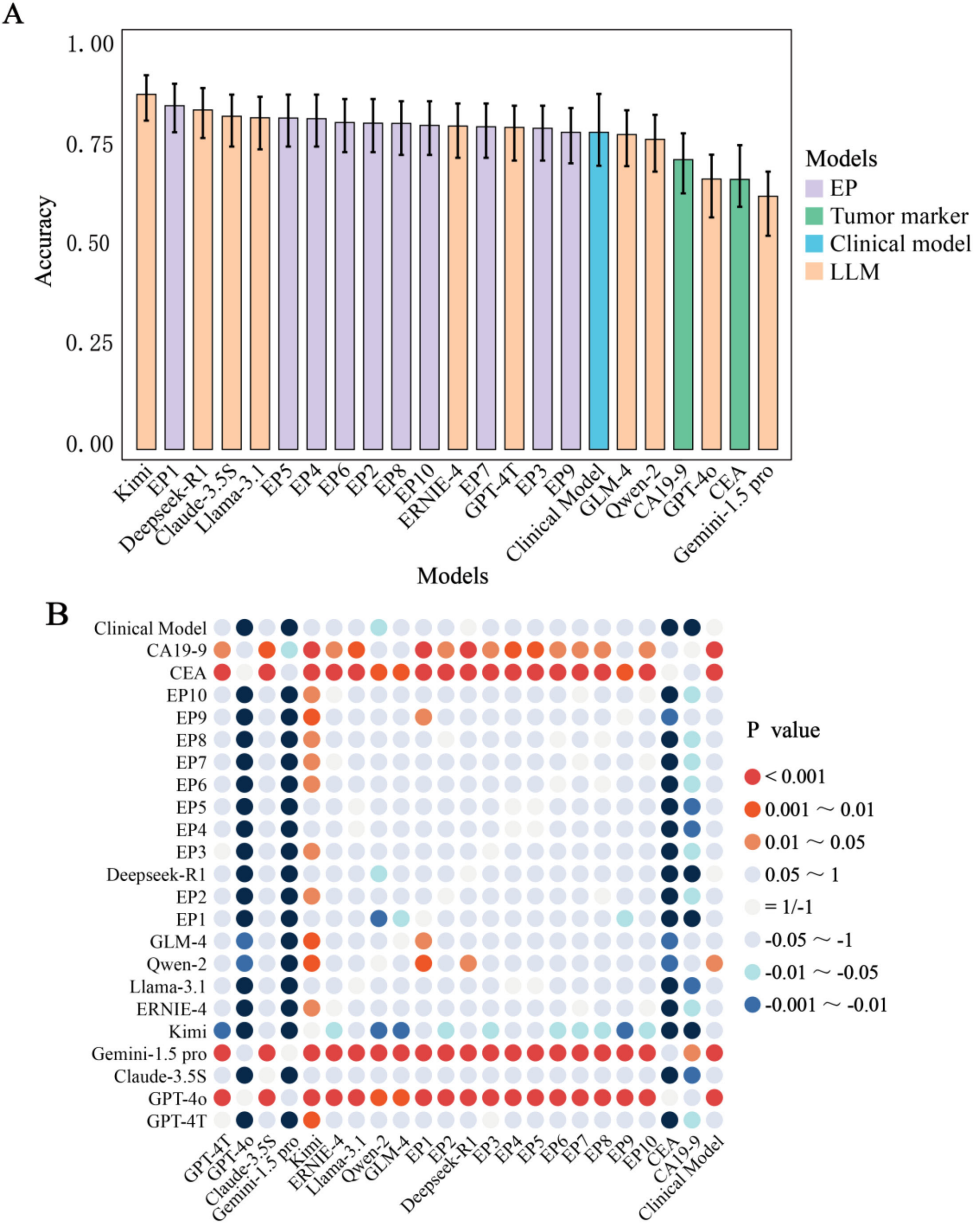
Comparative performance evaluation. **(A)** Accuracy among Different LLMs, Experienced Physicians, Clinical Model and Tumor Markers. **(B)** A Comparative Analysis of Diagnostic Accuracy and Significance Testing among models. The p-values (Holm-adjusted) were from the comparison of accuracy between the predictive groups listed along the horizontal axis and those on the vertical axis. A positive p-value indicates that the accuracy of the group on the horizontal axis is statistically greater than that of the group on the vertical axis, whereas a negative p-value signifies the opposite. CA19-9, Carbohydrate Antigen 199; CEA, Carcinoembryonic Antigen; EP, Experienced physician.

Four LLMs (40%), one clinical model, and six physicians (60%) achieved accuracies ≥80%. Kimi demonstrated the highest accuracy (87%), significantly outperforming 70% of physicians (7/10, p < 0.01) (**Table 3**, **Figure 3**, **Supplementary Table S2**). Top-performing LLMs including Deepseek-R1 (0.83), Claude-3.5S (0.82), Llama-3.1 (0.81), and GPT-4T (0.79) showed comparable accuracy to physicians (0.78–0.84, p > 0.05), while physicians outperformed lower-performing LLMs (GPT-4o, Gemini-1.5-pro). Eighty percent of LLMs exceeded CEA (0.66) and CA19-9 (0.71) in accuracy, with the clinical model (0.83) competing with top LLMs.

CA19–9 exhibited the highest sensitivity (0.93) across 23 predictive groups, significant in 18 groups (p: 0–0.044). GPT-4T, GPT-4o, and Gemini-1.5-pro showed the highest specificity (0.99), significant in 14 groups (p: 0–0.048). Kimi led in F1-score (0.87, significant in 12 groups), GPT-4T in PPV (0.98, 13 groups), and EP1 in NPV (0.85, 12 groups). Top LLMs dominated four metrics (accuracy, specificity, F1-score, PPV), while tumor markers and physicians excelled in sensitivity and NPV, respectively.

Analysis of 360 incorrect diagnoses revealed that over 80% resulted from LLMs over-relying on single data sources (**Supplementary Table S6**). Representative examples: Claude 3.5 misdiagnosed a benign stricture as malignant solely based on elevated CA19-9, while GPT-4T correctly integrated bilirubin trends, imaging, and histology (**Supplementary Material**).

Analysis of the misdiagnoses revealed >80% originated from LLMs over-relying on single data sources (**Supplementary Table S3**). For instance, Claude 3.5 misdiagnosed a benign stricture as malignant based solely on elevated CA19-9, whereas GPT-4T integrated bilirubin trends, imaging, and histology for correct diagnosis (**Supplementary Document**).

### Subgroup analysis by stricture location

**Figure 4:**
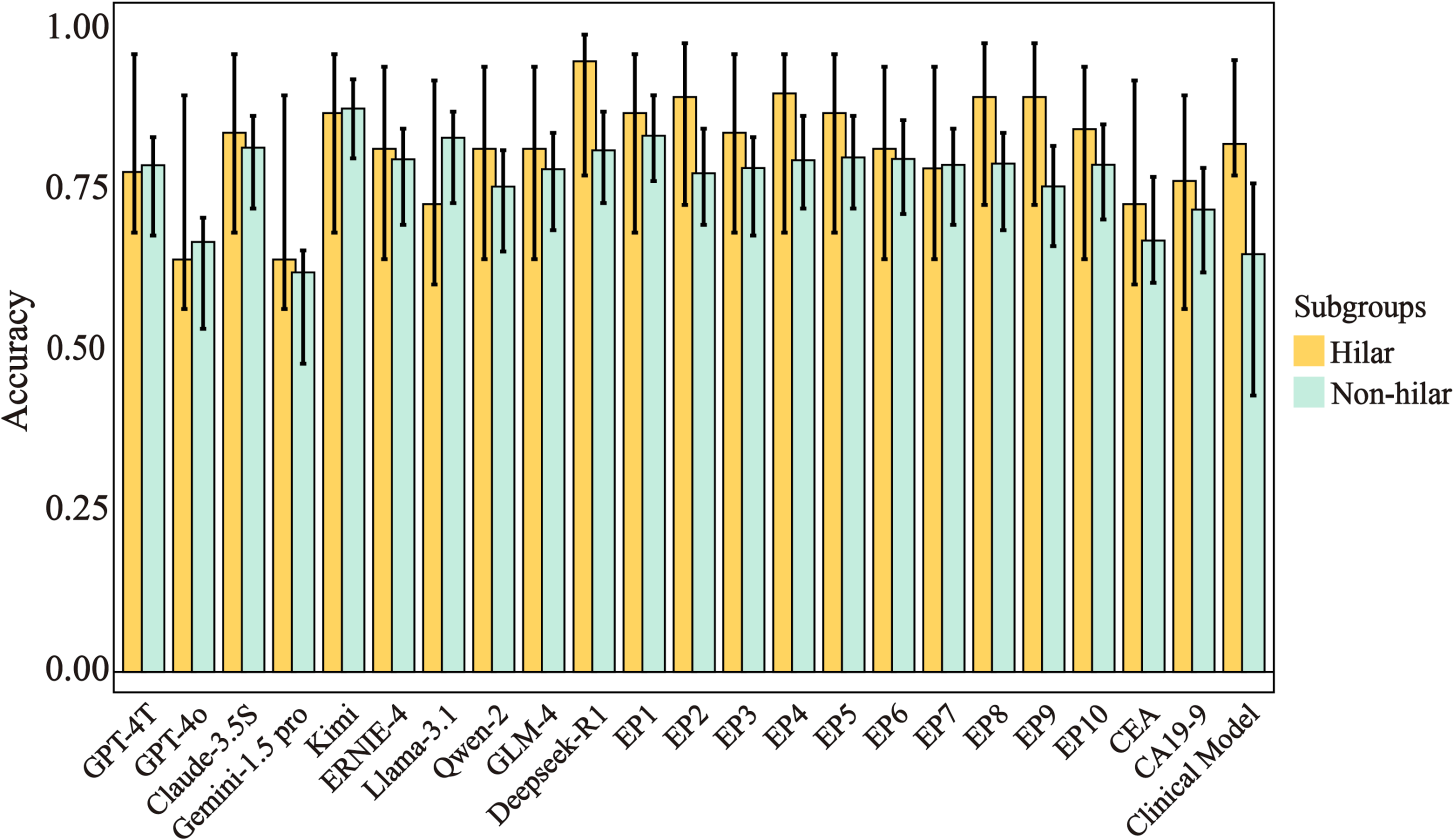
Accuracy and 95% CIs across subgroups for different prediction models. Error bars represent 95% confidence intervals. Hilar strictures (n=29: 9 benign, 20 malignant), non-hilar strictures (n=130: 74 benign, 56 malignant). CA19-9, Carbohydrate Antigen 199; CEA, Carcinoembryonic Antigen; EP, Experienced physician.

Performance metrics for hilar (n=29, 9 benign/20 malignant) and non-hilar subgroups are detailed in **Figure 4** and **Supplementary Table S4**. Given the limited sample size of hilar strictures (n=29, including 9 benign and 20 malignant cases), the subgroup analysis should be interpreted with caution due to potential overfitting risks. Despite this limitation, we observed that in Physicians showed higher accuracy in hilar versus non-hilar strictures (0.87 vs. 0.79, p < 0.001), while LLMs had comparable accuracy in both subgroups (hilar: 0.64-0.95; non-hilar: 0.62-0.88, p > 0.05). In the hilar subgroup, Deepseek-R1 showed highest hilar accuracy (0.95, 95% CI:0.77-0.99), followed by Kimi (0.87) and Claude-3.5S (0.84). Notably in this exploratory analysis, 7/10 physicians achieved superior accuracy over 8/10 LLMs (87-90% vs. 64-84%, p<0.05). In the non-hilar subgroup, LLMs showed competitive/exceeding accuracy. Given the small sample size, these findings should be interpreted as preliminary and require validation in larger cohorts.

### LLM diagnostic concordance assessment

Deepseek-R1, GPT-4T, GPT-4o, Llama-3.1, Gemini-1.5-pro, and Claude-3.5S showed near-perfect internal concordance (κ=0.81-0.97). Kimi, ERNIE-4, and GLM-4 demonstrated substantial concordance, while Qwen-2 showed fair concordance. When compared to true values, no model reached almost perfect concordance: Deepseek-R1, Llama-3.1, Claude-3.5S, and Kimi showed substantial concordance; ERNIE-4, GLM-4, Qwen-2 moderate; Gemini-1.5 pro and GPT-4o fair (**Supplementary Table S6**, **Supplementary Figure S3**).

## Discussion

The differentiation between benign and malignant biliary strictures remains clinically challenging. Current endoscopic techniques show limited sensitivity: ERCP-based brushing/biopsy (0.45-0.67) (27–29) and cholangioscopy biopsy (0.43-0.74) (2, 30) often prove inadequate. Our study demonstrates that select large language models (LLMs) achieve diagnostic accuracy rivaling or exceeding human expertise. Kimi attained the highest accuracy (87%), significantly outperforming 70% of experienced physicians (7/10, p<0.01). Three additional LLMs (Deepseek-R1:83%, Claude-3.5S:82%, Llama-3.1:81%) and our clinical prediction model (83%) performed comparably to physicians (78-84%, p>0.05). Collectively, 80% of LLMs surpassed conventional tumor markers (CA19–9 accuracy:66%; CEA:71%).

Notably, this is the first study to demonstrate that select large language models (LLMs) match or exceed the accuracy of a clinical model and experienced physicians. These findings suggest LLMs could serve as accessible, real-time diagnostic aids, particularly in resource-constrained settings where specialist expertise is limited. The variation among LLM performance reveals clinically meaningful insights. While GPT-4o and Gemini-1.5-Pro lead general benchmarking tasks (31), these models underperformed in our specific diagnostic application (accuracies 0.66 and 0.62 respectively). These underperforming models exhibited extreme specificity (0.99) and PPV (0.95-0.97) but critically low sensitivity (0.25-0.34), likely reflecting excessive safety prioritization during training protocols. This pattern necessitates caution when using such models for biliary stricture assessment. Conversely, Kimi’s superior performance (87%) highlights how task-specific optimization can yield exceptional diagnostic capability irrespective of general benchmarking performance.

Our subgroup analysis revealed physicians outperformed LLMs in the evaluation of hilar strictures (n=29), with 7/10 physicians achieving significantly higher accuracy than 8/10 LLMs (87-90% vs. 64-84%, p<0.05). This finding aligns with established clinical knowledge that >90% of hilar strictures are malignant (1), suggesting experienced clinicians better integrate this epidemiological context. The performance gap may indicate incomplete learning of clinical nuances by current LLMs. However, targeted fine-tuning with medical knowledge or Retrieval-Augmented Generation (RAG) (32–34) could potentially bridge this gap in future iterations. Clinically, this underscores the continued value of expert judgment in anatomically complex presentations, while suggesting LLMs may currently serve best as diagnostic aids for non-hilar strictures where they demonstrated parity with physicians. However, due to due to small sample size in the subgroup of hilar strictures, this analysis is exploratory and requires validation.

Our clinical prediction model, incorporating established risk factors (age, CA19-9, CEA, disease duration <1 month, CRP, surgical history, lymphadenopathy), achieved an AUC of 0.83 (95% CI:0.70-0.96), aligning with prior reports (AUC 0.75-0.83) (35–38) while maintaining practical clinical utility. CA19–9 demonstrated an expected AUC (0.77) matching literature reports (0.759-0.783) (35, 38), but presented a diagnostic paradox with high sensitivity (0.93) yet poor specificity (0.39). This suggests potential utility as a rule-out screening tool requiring subsequent confirmation, while CEA demonstrated weaker discriminative capacity (AUC 0.66) than some prior studies (39–42), emphasizing context-dependent variability.

Despite promising diagnostic capabilities, clinical implementation of LLMs faces significant barriers requiring strategic resolution. Error analysis demonstrated that >80% of misdiagnoses originated from LLMs over-relying on isolated data elements rather than multimodal integration. Representative examples included Claude 3.5 misclassifying a benign stricture as malignant based solely on elevated CA19-9, contrasting with GPT-4T’s accurate diagnosis achieved through synthesizing bilirubin trends, imaging findings, and histology. This significant limitation persists despite recent demonstrations of LLMs outperforming physicians in controlled diagnostic settings (43), underscoring a critical challenge in translating artificial intelligence capabilities to clinical practice where multimodal reasoning is essential. Text-based implementation currently constrains LLMs, but emerging multimodal capabilities in radiology (44–46) and dermatology (47) suggest promising diagnostic extensions. Future integration of CT/MRCP imaging could substantially enhance biliary stricture evaluation, though diagnosing rare conditions requires specialized training approaches. To address these constraints, we propose a structured workflow comprising: 1) electronic Medical Record-integrated real-time malignancy probability scoring, and 2) automatic referral to targeted multidisciplinary review for cases with LLM confidence scores below 80%. This structure preserves physician oversight while optimizing diagnostic efficiency, particularly valuable in resource-limited settings. However, clinical deployment of LLMs demands addressing several critical ethical considerations: 1) Accountability through legal frameworks addressing liability for diagnostic errors; 2) Hallucination mitigation requiring detection protocols (evidenced in 12% of erroneous outputs); 3) Patient acceptance considerations, with survey data showing 67% rejection of AI-exclusive diagnoses for cancer-related decisions; 4) Equity concerns including documented performance disparities in elderly populations; 5) Transparency requirements for interpretable decision pathways; and 6) Privacy mandates demanding robust data anonymization. Essential mitigation strategies include human-AI collaborative diagnostic models, algorithmic bias correction techniques, and targeted patient education initiatives clarifying LLMs’ assistive role.

Our study exhibits several limitations that warrant consideration. First, the retrospective single-center design introduces potential selection bias. Second, modest sample size limiting statistical power to address heterogeneity in biliary stricture presentations, potentially restricting generalizability across diverse healthcare settings; Third, the small hilar stricture subgroup (n = 29) limits statistical power for physician-LLM comparisons in this anatomically complex subset. Fourth, version-specific LLM evaluation restricts generalizability to updated iterations. Fifth, variability in physician experience levels may impact human performance benchmarks. Sixth, absence of external validation constrains generalizability.

## Conclusion

In conclusion, this study demonstrates select LLMs (Kimi, Deepseek-R1, Claude-3.5S, Llama-3.1) achieve diagnostic accuracy comparable to or exceeding clinical models and physicians for biliary strictures, though hilar cases remain challenging. Their optimal implementation involves augmenting clinical judgment rather than replacing it, especially valuable for non-hilar strictures where performance matched physicians.

## Data Availability

The original contributions presented in the study are included in the article/**Supplementary Material**. Further inquiries can be directed to the corresponding authors.
