# Supplementary material for: Performance of large language models in the differential diagnosis of benign and malignant biliary stricture
Source: Front Oncol. 2025 Jul 3;15:1613818. doi: 10.3389/fonc.2025.1613818 (PMC12267023; doi:10.3389/fonc.2025.1613818)
Supplement: Supplementary file 1 [file DataSheet1.docx]

**Supplemental** **Material**

**Title: Performance of Large Language Models in the Differential Diagnosis of Benign and Malignant Biliary Stricture**

**Supplementary Table 1:** Overview of Nine LLMs Utilized for Differential Diagnosis of Benign and Malignant Biliary Strictures

**Supplementary Table 2:** A Comparative Analysis of Diagnostic Accuracy and Significance Testing among LLMs, Clinical Model, and Experienced Physicians

**Supplementary Table 3:** LLMs’ Error Type Classification

**Supplementary Table 4:** Performance Metrics of Different Prediction Models in Hilar and Non-hilar Subgroups

**Supplementary Table 5:** Diagnostic Performance of Nine LLMs in Patients with Biliary Stricture (Rounds 1 & 2)

**Supplementary Table 6:** LLM Agreement in Predicting Benign versus Malignant Bile Duct Stricture

**Supplementary Figure 1:** Optimization of Hyperparameter λ for L2 Regularization in the Newly Developed Clinical Model: Accuracy Curve Derived from 10-fold Cross-validation

**Supplementary Figure 2:** Estimation of AUC Value and its 95% CI for ROC Curve of Newly Developed Clinical Model Using Bootstrap Method with 1000 Iterations

**Supplementary Figure 3:** Consistency Analysis of Nine LLMs

**Supplementary Method 1:** Inputting Prompts for Large Language Models

**Supplementary Document:** Analysis of LLM Reasoning Processes

**Supplementary Table 1: Overview of Nine LLMs Utilized for Differential Diagnosis of Benign and Malignant Biliary Strictures**

| LLMs | Version | Time | Developer | Country | website | Open-Source | Input Modality |
| --- | --- | --- | --- | --- | --- | --- | --- |
| GPT-4T | GPT-4.0-Turbo-128k | 2024/11 | OpenAI | USA | https://openai.com/index/gpt-4/ | No | Multimodal |
| GPT-4o | GPT-4o | 2024/11 | OpenAI | USA | https://openai.com/index/hello-gpt-4o/ | No | Multimodal |
| Claude-3.5S | Claude-3.5-Sonnet | 2024/11 | Anthropic | USA | https://claude.ai/ | No | Multimodal |
| Gemini-1.5 pro | Gemini-1.5-pro | 2024/11 | Google | USA | https://deepmind.google/technologies/gemini/pro/ | No | Multimodal |
| Kimi | Based on the version as of November, 2024 | 2024/11 | Moonshot AI | China | https://kimi.moonshot.cn/ | Yes | Multimodal |
| ERNIE-4 | ERNIE-4.0-Turbo-8K | 2024/11 | Baidu Inc. | China | https://yiyan.baidu.com/ | Yes | Multimodal |
| Llama-3.1 | Llama-3.1-405B | 2024/11 | Meta | USA | https://github.com/meta-llama | Yes | Text-only |
| Qwen-2 | Qwen-2-72B | 2024/11 | Alibaba Cloud | China | https://tongyi.aliyun.com/qianwen/ | Yes | Text-only |
| GLM-4 | GLM-4-9B | 2024/11 | Tsinghua University KEG Lab and Zhipu AI | China | https://chatglm.cn/ | Yes | Multimodal |
| Deepseek | Deepseek-R1 | 2025/2 | Deepseek | China | https://deepseek.com/ | Yes | Text-only |
| LLM: Large Language Model  All queries utilized identical deterministic parameters: temperature=0.0 (to ensure output consistency), max tokens=10240, and identical system prompts. | | | | | | | |

The table provides an overview of the nine LLMs used in our study for differential diagnosis of the benign and malignant nature of biliary strictures. It includes essential details such as the names, versions, release dates, developers, websites, and countries of origin of the models.

**Supplementary Table 2: A Comparative Analysis of Diagnostic Accuracy and Significance Testing among LLMs, Clinical Model, and Experienced Physicians**


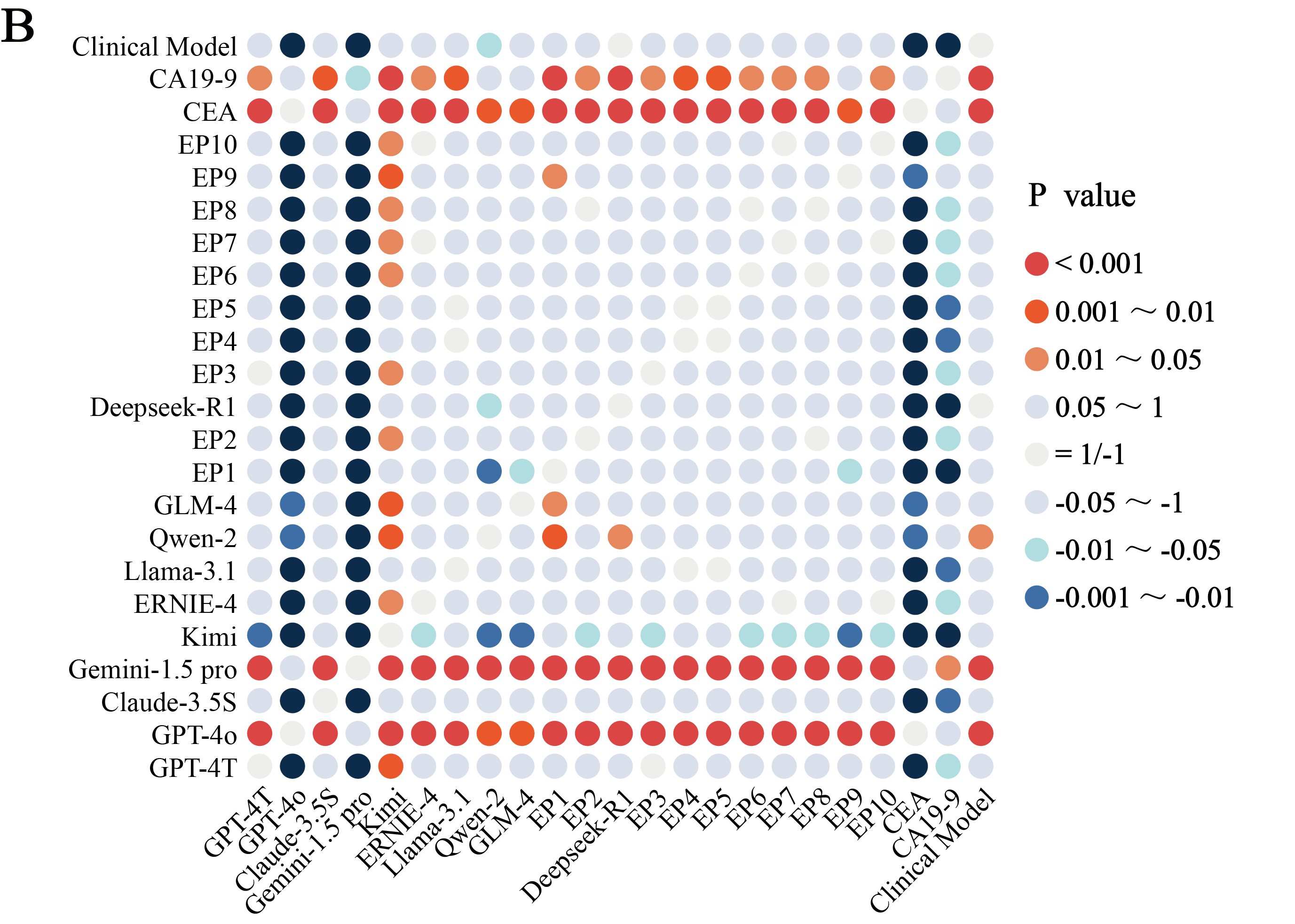


EP: Experienced Physician; CA19-9: Carbohydrate Antigen 199; CEA: Carcinoembryonic Antigen

To evaluate the statistical significance of differences in diagnostic performance, we employed the McNemar Test (with Holm-Bonferroni correction), which is a non-parametric statistical method specifically designed for paired nominal data. This test allows us to assess whether the observed differences in diagnostic accuracy between any two groups are statistically significant, thereby providing robust evidence for the comparative analysis presented in Supplementary Table 2.

**Supplementary Table 3: LLMs’ Error Type Classification**

| **LLMs** | **Incorrect Cases** | **Judgment Relying on Single Abnormal Information** |
| --- | --- | --- |
| GPT-4T | 33 | 30 (90.9%) |
| GPT-4o | 54 | 50 (92.6%) |
| Claude-3.5S | 29 | 24 (82.8%) |
| Gemini-1.5 pro | 60 | 52 (86.7%) |
| Kimi | 20 | 16 (80.0%) |
| Llama-3.1 | 33 | 29 (87.9%) |
| ERNIE-4 | 30 | 27 (90.0%) |
| Qwen-2 | 38 | 31 (81.6%) |
| GLM-4 | 36 | 30 (83.3%) |
| Deepseek-R1 | 27 | 24 (88.9%) |
| Note: "Errors Due to Single Information Source Reliance" refers to cases where LLMs made incorrect judgments by over-relying on isolated abnormal information while neglecting contextual relevance or multi-source information integration. It presents the classification of error types in LLMs, displaying the number of errors for different LLMs and the proportion of errors caused by judgments relying on single information sources. | | |

**Supplementary Table 4: Performance Metrics of Different Prediction Models in Hilar and Non-hilar Subgroups**

| **Subgroup** | **Models** | **Accuracy**  **(95%CI)** | **Sensitivity**  **(95%CI)** | **Specificity**  **(95%CI)** | **F1 score** | **PPV** | **NPV** |
| --- | --- | --- | --- | --- | --- | --- | --- |
| Hilar | GPT-4o | 0.64 (0.56, 0.90) | 0.33 (0.21, 0.49) | 0.95 (0.89, 0.98) | 0.46 | 0.75 | 0.76 |
|  | GPT-4T | 0.78 (0.68, 0.96) | 0.56 (0.42, 0.66) | 1.00 (0.91, 1.00) | 0.71 | 1.00 | 0.83 |
|  | Claude-3.5S | 0.84 (0.68, 0.96) | 0.78 (0.61, 0.83) | 0.90 (0.83, 0.93) | 0.78 | 0.78 | 0.90 |
|  | Gemini-1.5 pro | 0.64 (0.56, 0.90) | 0.33 (0.25, 0.44) | 0.95 (0.84. 0.99) | 0.46 | 0.75 | 0.76 |
|  | Kimi | 0.87 (0.68, 0.96) | 0.89 (0.78, 0.92) | 0.85 (0.77, 0.93) | 0.80 | 0.73 | 0.94 |
|  | ERNIE-4 | 0.81 (0.64, 0.94) | 0.78 (0.70, 0.86) | 0.85 (0.75, 0.93) | 0.74 | 0.70 | 0.89 |
|  | Llama-3.1 | 0.73 (0.60, 0.92) | 0.56 (0.51, 0.69) | 0.90 (0.81, 0.95) | 0.63 | 0.71 | 0.82 |
|  | Qwen-2 | 0.81 (0.64, 0.94) | 0.78 (0.70, 0.83) | 0.85 (0.78, 0.93) | 0.74 | 0.70 | 0.89 |
|  | GLM-4 | 0.81 (0.64, 0.94) | 0.78 (0.71, 0.83) | 0.85 (0.79, 0.92) | 0.74 | 0.70 | 0.89 |
|  | Clinical Model | 0.89 (0.77, 0.99) | 0.78 (0.71, 0.85) | 1.00 (0.93, 1.00) | 0.88 | 1.00 | 0.91 |
|  | Deepseek-R1 | 0.95(0.77, 0.99) | 1.00 (0.91, 1.00) | 0.90 (0.83, 0.95) | 0.90 | 0.82 | 1.00 |
|  | EP1 | 0.87 (0.68, 0.96) | 0.89 (0.80. 0.93) | 0.85 (0.79, 0.94) | 0.80 | 0.73 | 0.94 |
|  | EP2 | 0.89 (0.73, 0.98) | 0.89 (0.82, 0.95) | 0.90 (0.81, 0.96) | 0.84 | 0.80 | 0.95 |
|  | EP3 | 0.84 (0.68, 0.96) | 0.78 (0.66, 0.83) | 0.90 (0.83, 0.95) | 0.78 | 0.78 | 0.90 |
|  | EP4 | 0.90 (0.68, 0.96) | 1.00 (0.93, 1.00) | 0.80 (0.72, 0.88) | 0.82 | 0.69 | 1.00 |
|  | EP5 | 0.87 (0.68, 0.96) | 0.89 (0.81, 0.94) | 0.85 (0.78, 0.92) | 0.80 | 0.73 | 0.94 |
|  | EP6 | 0.81 (0.64, 0.94) | 0.78 (0.70, 0.85) | 0.85 (0.78, 0.95) | 0.74 | 0.70 | 0.89 |
|  | EP7 | 0.78 (0.64, 0.94) | 0.67 (0.60, 0.78) | 0.90 (0.85, 0.94) | 0.71 | 0.75 | 0.86 |
|  | EP8 | 0.89 (0.73, 0.98) | 0.89 (0.81, 0.95) | 0.90 (0.84, 0.96) | 0.84 | 0.80 | 0.95 |
|  | EP9 | 0.89 (0.73, 0.98) | 0.89 (0.81, 0.95) | 0.90 (0.82, 0.96) | 0.84 | 0.80 | 0.95 |
|  | EP10 | 0.84 (0.64, 0.94) | 0.89 (0.80, 0.95) | 0.80 (0.72, 0.86) | 0.76 | 0.67 | 0.94 |
|  | CEA | 0.73 (0.60, 0.92) | 0.56 (0.50 0.62) | 0.90 (0.82, 0.96) | 0.63 | 0.71 | 0.82 |
|  | CA19-9 | 0.76 (0.56, 0.90) | 0.78 (0.70, 0.88) | 0.75 (0.69, 0.81) | 0.67 | 0.58 | 0.88 |
| Non-Hilar | GPT-4o | 0.67 (0.53, 0.71) | 0.34 (0.29, 0.39) | 1.00 (0.92, 1.00) | 0.51 | 1.00 | 0.53 |
|  | GPT-4T | 0.79 (0.68, 0.83) | 0.59 (0.50, 0.65) | 0.98 (0.92, 1.00) | 0.74 | 0.98 | 0.65 |
|  | Claude-3.5S | 0.82 (0.72, 0.86) | 0.70 (0.66, 0.76) | 0.93 (0.88, 0.96) | 0.80 | 0.93 | 0.70 |
|  | Gemini-1.5 pro | 0.62 (0.48, 0.66) | 0.24 (0.20, 0.31) | 1.00 (0.93, 1.00) | 0.39 | 1.00 | 0.50 |
|  | Kimi | 0.88 (0.80, 0.92) | 0.82 (0.75, 0.88) | 0.93 (0.86, 0.97) | 0.88 | 0.94 | 0.80 |
|  | ERNIE-4 | 0.80(0.70, 0.85) | 0.65 (0.60, 0.71) | 0.95 (0.90, 0.98) | 0.77 | 0.94 | 0.67 |
|  | Llama-3.1 | 0.83(0.73, 0.87) | 0.66 (0.58, 0.71) | 1.00 (0.91, 1.00) | 0.80 | 1.00 | 0.69 |
|  | Qwen-2 | 0.76(0.65, 0.81) | 0.64 (0.57, 0.70) | 0.88 (0.81, 0.92) | 0.73 | 0.87 | 0.64 |
|  | GLM-4 | 0.78(0.69, 0.84) | 0.69 (0.61, 0.75) | 0.88 (0.80, 0.93) | 0.77 | 0.88 | 0.68 |
|  | Clinical Model | 0.65(0.43, 0.76) | 0.36 (0.30, 0.42) | 0.94 (0.89, 0.98) | 0.52 | 0.89 | 0.52 |
|  | Deepseek-R1 | 0.81(0.73, 0.87) | 0.78 (0.70, 0.85) | 0.84 (0.78, 0.89) | 0.82 | 0.87 | 0.75 |
|  | EP1 | 0.83(0.76, 0.90) | 0.86 (0.80, 0.92) | 0.80 (0.72, 0.88) | 0.86 | 0.85 | 0.82 |
|  | EP2 | 0.78(0.70, 0.85) | 0.78 (0.71, 0.85) | 0.77 (0.71, 0.83) | 0.80 | 0.82 | 0.73 |
|  | EP3 | 0.78(0.68, 0.83) | 0.62 (0.55, 0.69) | 0.95 (0.91, 0.98) | 0.75 | 0.94 | 0.65 |
|  | EP4 | 0.80(0.72, 0.86) | 0.82 (0.78, 0.88) | 0.77 (0.73, 0.83) | 0.82 | 0.82 | 0.77 |
|  | EP5 | 0.80(0.72, 0.86) | 0.80 (0.74, 0.85) | 0.80 (0.76, 0.84) | 0.82 | 0.84 | 0.75 |
|  | EP6 | 0.80(0.71, 0.86) | 0.76 (0.70, 0.82) | 0.84 (0.79, 0.89) | 0.81 | 0.86 | 0.72 |
|  | EP7 | 0.79(0.70, 0.85) | 0.70 (0.63, 0.75) | 0.88 (0.82, 0.93) | 0.78 | 0.88 | 0.69 |
|  | EP8 | 0.79(0.69, 0.84) | 0.64 (0.58, 0.73) | 0.95 (0.90, 0.99) | 0.76 | 0.94 | 0.66 |
|  | EP9 | 0.76(0.66, 0.82) | 0.69 (0.64, 0.75) | 0.82 (0.78, 0.88) | 0.76 | 0.84 | 0.67 |
|  | EP10 | 0.79(0.70, 0.85) | 0.76 (0.70, 0.82) | 0.82 (0.78, 0.89) | 0.80 | 0.85 | 0.72 |
|  | CEA | 0.67(0.61, 0.77) | 0.82 (0.76, 0.89) | 0.52 (0.48, 0.59) | 0.75 | 0.69 | 0.69 |
|  | CA19-9 | 0.72(0.62, 0.78) | 0.64 (0.58, 0.71) | 0.80 (0.74, 0.85) | 0.71 | 0.81 | 0.63 |

CA19-9: Carbohydrate Antigen 199; CEA: Carcinoembryonic Antigen; EP: Experienced physician.

**Supplementary Table 5:** **Diagnostic Performance of Nine LLMs in Patients with Biliary Stricture (Rounds 1 & 2)**

| LLMs | Balanced accuracy | Sensitivity | Specificity | F1 score | PPV | NPV |
| --- | --- | --- | --- | --- | --- | --- |
| GPT-4T R1 | 0.79 (0.71, 0.84) | 0.59 (0.52, 0.65) | 0.99 (0.91, 1.00) | 0.74 | 0.98 | 0.69 |
| GPT-4T R2 | 0.77 (0.69, 0.82) | 0.65 (0.60, 0.71) | 0.88 (0.82, 0.94) | 0.74 | 0.86 | 0.70 |
| GPT-4o R1 | 0.66 (0.57, 0.72) | 0.34 (0.29, 0.39) | 0.99 (0.92, 1.00) | 0.50 | 0.97 | 0.58 |
| GPT-4o R2 | 0.64 (0.54, 0.70) | 0.29 (0.25, 0.34) | 0.99 (0.94, 1.00) | 0.44 | 0.96 | 0.56 |
| Claude-3.5S R1 | 0.82 (0.74, 0.87) | 0.71 (0.66, 0.77) | 0.92 (0.88, 0.95) | 0.80 | 0.91 | 0.74 |
| Claude-3.5S R2 | 0.81 (0.73, 0.86) | 0.70 (0.72, 0.78) | 0.92 (0.87, 0.96) | 0.79 | 0.91 | 0.74 |
| Gemini-1.5 pro R1 | 0.62 (0.52, 0.68) | 0.25 (0.20, 0.31) | 0.99 (0.94, 1.00) | 0.40 | 0.95 | 0.55 |
| Gemini-1.5 pro R2 | 0.61 (0.52, 0.67) | 0.23 (0.18, 0.26) | 1.00 (0.93, 1.00) | 0.37 | 1.00 | 0.54 |
| Kimi R1 | 0.87 (0.81, 0.92) | 0.83 (0.78, 0.89) | 0.91 (0.86, 0.96) | 0.87 | 0.91 | 0.83 |
| Kimi R2 | 0.85 (0.78, 0.90) | 0.81 (0.76, 0.88) | 0.89 (0.84, 0.95) | 0.85 | 0.89 | 0.81 |
| Llama-3.1 R1 | 0.79 (0.71, 0.85) | 0.63 (0.58, 0.69) | 0.96 (0.90, 0.98) | 0.75 | 0.95 | 0.70 |
| Llama-3.1 R2 | 0.81 (0.73, 0.86) | 0.65 (0.60, 0.71) | 0.97 (0.92, 0.99) | 0.78 | 0.96 | 0.72 |
| ERNIE-4 R1 | 0.79 (0.71, 0.85) | 0.66 (0.60, 0.71) | 0.92 (0.88, 0.96) | 0.76 | 0.90 | 0.71 |
| ERNIE-4 R2 | 0.75 (0.67, 0.81) | 0.54 (0.49, 0.59) | 0.96 (0.90, 0.99) | 0.69 | 0.94 | 0.66 |
| Qwen-2 R1 | 0.69 (0.61, 0.76) | 0.63 (0.58, 0.69) | 0.76 (0.70, 0.82) | 0.68 | 0.74 | 0.65 |
| Qwen-2 R2 | 0.76 (0.68, 0.82) | 0.65 (0.61, 0.71) | 0.87 (0.82, 0.93) | 0.73 | 0.84 | 0.69 |
| GLM-4 R1 | 0.77 (0.69, 0.83) | 0.69 (0.63, 0.75) | 0.86 (0.81, 0.92) | 0.75 | 0.84 | 0.71 |
| GLM-4 R2 | 0.77 (0.69, 0.83) | 0.67 (0.61, 0.75) | 0.87 (0.82, 0.93) | 0.75 | 0.85 | 0.71 |
| Deepseek-R1 R1 | 0.83 (0.76, 0.89) | 0.81 (0.75, 0.86) | 0.86 (0.79, 0.91) | 0.83 | 0.86 | 0.80 |
| Deepseek-R1 R2 | 0.82 (0.75, 0.87) | 0.80 (0.76, 0.85) | 0.87 (0.82, 0.93) | 0.84 | 0.86 | 0.81 |
| LLM: Large Language Models; PPV: Positive Predictive Value; NPV: Negative Predictive Value; CI: Confidence Interval; R: Round. | | | | | | |

The table outlines the diagnostic performance of nine LLMs in two rounds, evaluating balanced accuracy, sensitivity, specificity, F1-score, PPV, and NPV. Several LLMs, including Kimi, Claude-3.5S, Llama-3.1, and GPT-4T, demonstrated consistently high balanced accuracies.

**Supplementary Table 6: LLM Agreement in Predicting Benign versus Malignant Bile Duct Stricture**

| LLMs | Inter-LLM Agreement | LLM vs truth agreement |
| --- | --- | --- |
| GPT-4T | κ=0.86, [95%CI: 0.77, 0.92] | κ=0.57, [95%CI: 0.45, 0.67] |
| GPT-4o | κ=0.81, [95%CI: 0.72, 0.90] | κ=0.36, [95%CI: 0.27, 0.46] |
| Claude-3.5S | κ=0.97, [95%CI: 0.94, 1.00] | κ=0.62, [95%CI: 0.52, 0.72] |
| Gemini-1.5 pro | κ=0.94, [95%CI: 0.87, 0.99] | κ=0.26, [95%CI: 0.17, 0.36] |
| Kimi | κ=0.75, [95%CI: 0.65, 0.83] | κ=0.74, [95%CI: 0.65, 0.83] |
| Llama-3.1 | κ=0.89, [95%CI: 0.82, 0.94] | κ=0.61, [95%CI: 0.50, 0.70] |
| ERNIE-4 | κ=0.79, [95%CI: 0.70, 0.86] | κ=0.58, [95%CI: 0.47, 0.68] |
| Qwen-2 | κ=0.35, [95%CI: 0.22, 0.48] | κ=0.51, [95%CI: 0.40, 0.62] |
| GLM-4 | κ=0.72, [95%CI: 0.63, 0.80] | κ=0.56, [95%CI: 0.45, 0.65] |
| Deepseek-R1 | κ=0.92, [95%CI: 0.83, 0.98] | κ=0.65, [95%CI: 0.53, 0.76] |
| LLM: Large Language Models; CI: Confidence Interval.  κ values: 0.20 or lower indicates slight concordance, 0.21-0.40 indicates fair concordance, 0.41-0.60 indicates moderate concordance, 0.61-0.80 indicates substantial concordance, and 0.81-1.00 indicates almost perfect concordance. | | |

**Supplementary Figure 1: Optimization of Hyperparameter λ in the Clinical Model: Accuracy Curve Derived from 10-fold Cross-validation.**

AUC: Area Under the Curve.


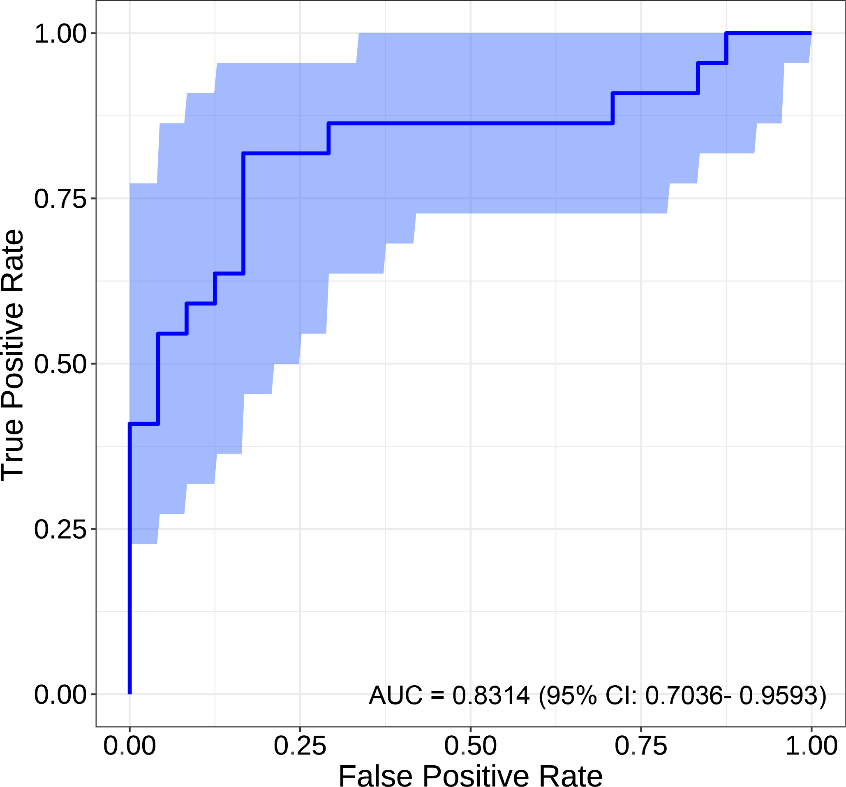


**Supplementary Figure 2: ROC Curve of Newly Developed Clinical Model.**

AUC: Area Under the Curve; CI: Confidence Interval.


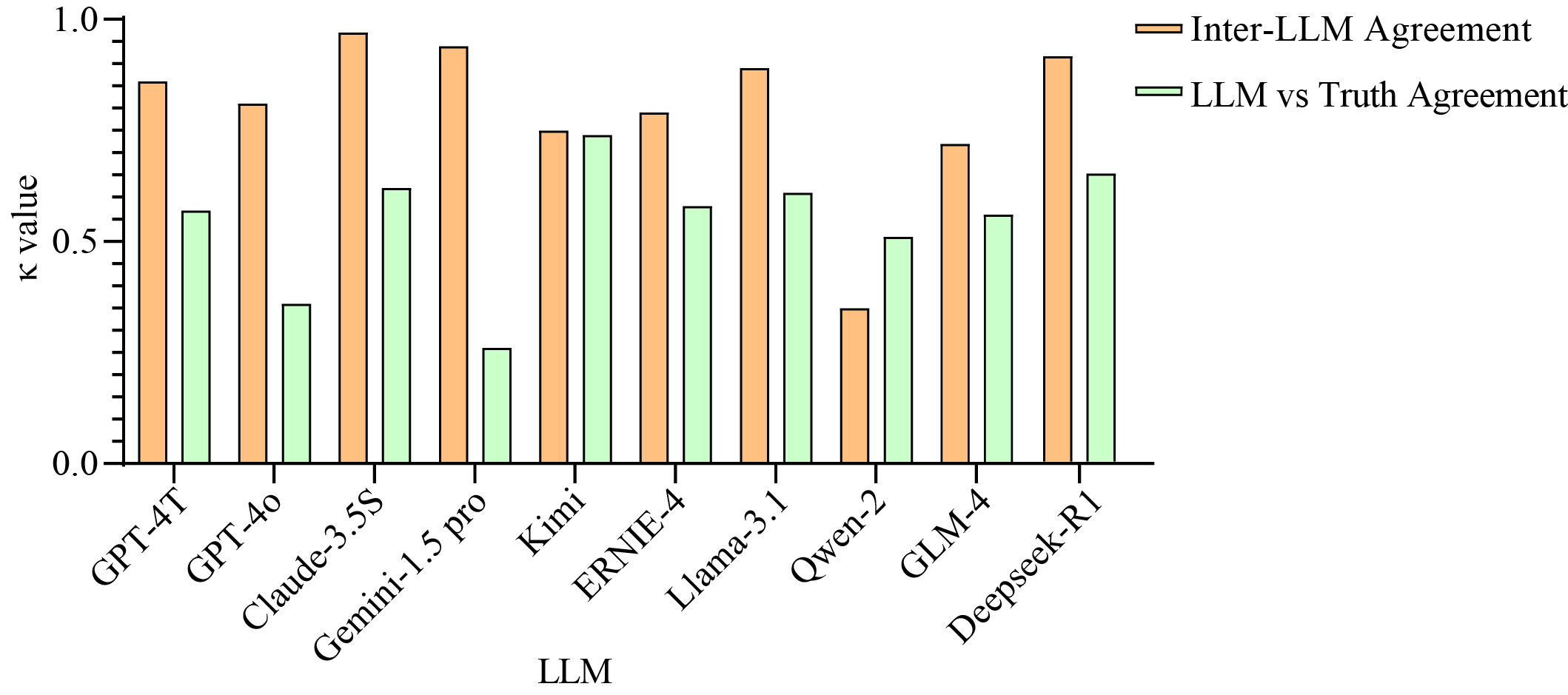


**Supplementary Figure 3: Concordance Analysis of Ten LLMs.**

LLM: Large Language Model.

The presented bar graph is a visual representation of the performance evaluation of 9 different LLMs. The horizontal axis of the graph displays the names of these Ten LLMs. The graph utilizes two distinct colors to distinguish between two types of consistency: one represents the consistency between the model's judgment results in two repetitions, and the other represents the consistency between the model's judgment results and the actual values.

**Supplementary Method 1: Inputting Prompts for Large Language Models**

**Chinese:**

“现在你作为一名胆胰疾病专科医生。

上传的文本文档是一个住院患者的病历信息，包含患者的住院病历记录、实验室检查结果、影像学描述结果，这是一名胆道狭窄的患者。

请根据病历中的信息，判断患者胆道狭窄的具体病因是良性还是恶性疾病。

给出倾向性判断，例如用“可能是”、“更可能是”、“倾向于是”等程度用词表示推断结果。”

**English:**

Now you are a specialist in biliary and pancreatic diseases.
The uploaded text document contains the medical records of an inpatient, including the patient's hospitalization records, laboratory test results, and imaging findings. This is a patient with biliary stricture.
Based on the information in the medical records, please determine whether the specific cause of the patient's biliary stricture is benign or malignant.
Provide a tentative judgment, using terms such as "likely," "more likely," or "tends to be" to indicate the inference.

**Supplementary Document: Analysis of LLM Reasoning Processes**

**Analysis Process of Incorrect Case 1:**

"Case1：1.患者老年男性，70岁，慢性病程。2.既往史：否认肝炎、结核、疟疾等传染病史，患有糖尿病6年余，未规律监测血糖，注射门冬胰岛素治疗（剂量不详），患有“病态窦房结综合征”，未予重视及诊治。否认“高血压”等病史，否认手术史，否认外伤史，否认输血史，否认药物、食物过敏史，预防接种史不详，否认中毒史。3.现病史：入院前1月余，患者无明显诱因出现乏力、食欲减退，就诊于外院检查提示转氨酶升高、肝功异常（具体不详），行相关检查后于2023年10月16日在宝鸡市人民医院行ERCP+ECT+ENBD+DSA术，诊断为“胆总管结石、十二指肠憩室”，给予保肝、抗感染治疗后好转出院。后上述症状反复发作，伴皮肤、巩膜黄染及尿黄，尿液颜色呈浓茶样，伴全身皮肤瘙痒，无恶心、呕吐，无头晕、头痛，无咳嗽、胸闷、气短等症状，就诊于宝鸡市人民医院，分别于2023年12月11日和2023年12月21行ERCP+EST+网篮取石、清理+ENBD术，术中诊断为“胆总管泥沙样结石、十二指肠憩室综合征”，给予对症治疗后好转。此次入院前10天，患者无明显诱因出现乏力、食欲减退，伴发热（未测体温），伴皮肤、巩膜黄染及尿黄，伴肝区胀满感，无明显恶心、呕吐，无呕血、黑便、便血等不适，遂就诊于我院急诊，行检验提示：丙氨酸氨基转移酶(ALT) 63IU/L↑，天门冬氨酸转氨酶(AST)88IU/L↑，碱性磷酸酶(ALP) 625IU/L↑，γ-谷氨酰基转移酶(GGT) 312IU/L↑，白蛋白/球蛋白(A/G) 0.8↓，钾(K) 3.47mmol/L↓，钠(Na) 133.1mmol/L↓，氯(CL) 98.0mmol/L↓，肌酐(CRE) 325μmol/L↑，白蛋白(ALB) 27.9g/L↓，总胆红素(TBIL) 408.5μmol/L↑，直接胆红素(DBIL) 375.1μmol/L↑，间接胆红素(IBIL) 33.40μmol/L↑，葡萄糖(GLU) 7.97mmol/L↑，总蛋白(TP) 62.5g/L↓。血常规：红细胞计数(RBC) 3.44X10E12/L↓，血红蛋白(HGB) 90g/L↓，血细胞比容(HCT) 0.278↓。血培养提示大肠埃希菌感染，药敏结果提示美罗培南敏感。MRCP示： 胆总管结石并管腔稍扩张；胆囊体积小、显示欠清晰，扫及腹水，请结合临床及腹部增强扫描。腹部CT示：1.右肺中叶、左肺上叶尖后段及双侧斜裂旁小结节，双肺下叶胸膜下少许索条。2.胆总管下段闭塞，待排占位或炎性，肝内外胆管明显扩张，请结合临床；十二指肠水平段致密影，请结合临床。双肾囊肿，右肾结石。今为进一步治疗来我院就诊，门诊以""1.梗阻性黄疸；2.急性胆管炎；3.脓毒症；4.肾功能不全；5.""收入院。自发病以来，患者精神尚可，体力正常，食欲正常，睡眠正常，体重无明显变化，大便正常，排尿正常。体重指数（BMI）:20.20 kg/m2。NRS评分 0分。4.查体：全身皮肤、巩膜黄染，左侧腹部可见PTCD管引流，引流出黄褐色液体约300ml，腹平坦，未见胃肠形及蠕动波，未见腹壁静脉曲张。全腹无明显压痛，肝曲无叩击痛，无反跳痛，无肌紧张，Murphy征阴性，全腹未扪及包块，肝、脾肋下未及。肝、肾区无叩击痛，腹部移动性浊音阴性。听诊肠鸣音正常。5.辅助检查：（2024年1月17日 本院）碱性磷酸酶(ALP) 625IU/L↑，γ-谷氨酰基转移酶(GGT) 312IU/L↑，白蛋白/球蛋白(A/G) 0.8↓，钾(K) 3.47mmol/L↓，钠(Na) 133.1mmol/L↓，氯(CL) 98.0mmol/L↓，肌酐(CRE) 325μmol/L↑，间接胆红素(IBIL) 33.40μmol/L↑，丙氨酸氨基转移酶(ALT) 63IU/L↑，天门冬氨酸转氨酶(AST)88IU/L↑，白蛋白(ALB) 27.9g/L↓，总胆红素(TBIL) 408.5μmol/L↑，直接胆红素(DBIL) 375.1μmol/L↑，葡萄糖(GLU) 7.97mmol/L↑，总蛋白(TP) 62.5g/L↓。血常规：红细胞计数(RBC) 3.44X10E12/L↓，血红蛋白(HGB) 90g/L↓，血细胞比容(HCT) 0.278↓。血培养提示大肠埃希菌感染，药敏结果提示美罗培南敏感。（2024年1月17日 本院）腹部CT提示：1.右肺中叶、左肺上叶尖后段及双侧斜裂旁小结节，建议随诊，双肺下叶胸膜下少许索条。2.胆总管下段闭塞，待排占位或炎性，肝内外胆管明显扩张，请结合临床；十二指肠水平段致密影，请结合临床。双肾囊肿，右肾结石。 （2024年1月23日 本院）MRCP提示：胆总管结石并管腔稍扩张，胆囊体积小、显示欠清晰，扫及腹水，请结合临床及腹部增强扫描。

影像学检查结果1：

CT 胆道增强+三维成像,三维专用

双侧胸腔积液，双肺下叶轻度膨胀不全。右肺中叶见实性小结节影。 胆总管末端软组织增厚，肝内胆管可见引流管，肝内外胆管见轻度扩张，肝外胆管壁毛糙，十二指肠水平段见结节状致密影并周围放射状伪影。 肝脏形态、大小正常，肝左叶见低密度无强化影。胆囊不大，壁未见明显增厚，囊腔内未见异常密度影。脾脏形态、大小正常，实质密度均匀，增强后未见明显异常强化灶。胰腺形态、大小未见异常，实质密度均匀，胰周脂肪间隙清晰。腹膜后未见明显肿大淋巴结影。腹腔少量积液。 双肾见小囊状无强化低密度影，右肾见径约0.4cm小结节状高密度影。右肾及上段输尿管轻度积水。

影像学检查结果2：

MR 胰胆管水成像(MRCP)(1.5T)

MRCP：肝内外胆管扩张不著，胆总管稍增宽，其内见低信号结节，较大0.6cm，胆囊体积小、显示欠清晰，胰管显影良好，未见明显扩张。扫及腹水。

异常实验室检查：

白细胞计数#：9.9 X10E9/L（参考值：3.50-9.50）

中性粒细胞百分率：0.797 （参考值：0.400-0.750）

淋巴细胞百分率：0.119 （参考值：0.200-0.500）

中性粒细胞绝对值：7.89 X10E9/L（参考值：1.80-6.30）

红细胞计数#：3.2 X10E12/L（参考值：4.30-5.80）

血红蛋白#：86 g/L（参考值：130-175）

血细胞比容#：0.269 （参考值：0.400-0.500）

平均血红蛋白含量：26.9 pg（参考值：27.0-34.0）

平均血红蛋白浓度：320 g/L（参考值：326-354）

总蛋白*：57.2 g/L（参考值：65.0-85.0）

白蛋白*：28.5 g/L（参考值：40.0-55.0）

总胆红素*：177.8 μmol/L（参考值：3.4-20.5）

直接胆红素：148.2 μmol/L（参考值：0.0-6.8）

间接胆红素：29.6 μmol/L（参考值：6.8-12.0）

碱性磷酸酶：220 IU/L（参考值：45-125）

γ-谷氨酰基转移酶：135 IU/L（参考值：10-60）

白蛋白/球蛋白：1 （参考值：1.2-2.4）

亮氨酸氨基肽酶：78 IU/L（参考值：30-70）

总胆汁酸：41.13 μmol/L（参考值：0.00-15.00）

甘油三脂*：5.74 mmol/L（参考值：<1.70）

高密度脂蛋白胆固醇*：0.62 mmol/L（参考值：0.91-2.06）

尿素*：34.66 mmol/L（参考值：3.60-9.50）

肌酐*：391 μmol/L（参考值：52-101）

尿酸：466 μmol/L（参考值：150-430）

胱抑素C：1.81 mg/L（参考值：0.51-1.09）

二氧化碳：13.4 mmol/L（参考值：20.0-29.0）

肌钙蛋白I：0.277 ng/ml（参考值：0-0.03）

B型尿钠肽检测（BNP）：720.9 pg/ml（参考值：<38.00）

心脏型脂肪酸结合蛋白：30.34 ng/ml（参考值：<6.20）

凝血酶原时间：16 s（参考值：7.7-10.5）

凝血酶时间：20.1 s（参考值：14.50-19.20）

D-二聚体：4.2 mg/L FEU（参考值：<0.50）

纤维蛋白原降解产物(血浆)：8.21 μg/mL（参考值：<5.00）

凝血酶原活动度：37.7 %（参考值：71.00-148.00）

PT国际标准化比值：1.74 （参考值：0.91-1.15）

C反应蛋白：150.1 mg/L（参考值：<10.0）

白介素6测定：249.5 pg/ml（参考值：<7.0）

降钙素原定量检测：30.12 ng/ml（参考值：<0.05）"

**Analysis Process 1:**

倾向性判断：胆道狭窄原因更可能为良性（如胆总管结石或炎性狭窄）

推理逻辑链

以下基于提供的病历资料，逐步分析胆道狭窄原因更倾向良性（而非恶性）的逻辑链。推理核心在于：患者有明确的胆石症病史、治疗响应、影像学特征及感染背景，这些更符合良性病因（如结石复发或炎性狭窄），而恶性线索（如年龄、软组织增厚）虽存在但较弱，且被良性因素覆盖。逻辑链分为关键步骤，引用病历证据支持。

起点：患者病史和既往治疗响应强烈支持良性病因

患者为70岁老年男性（年龄是恶性风险因素，但非决定性），病程反复但多次对结石治疗有效：

2023年10月、12月两次因类似症状（黄疸、肝功异常）行ERCP手术，均诊断为“胆总管结石”或“胆总管泥沙样结石”，并取出结石。治疗后症状好转（病历：“给予保肝、抗感染治疗后好转出院”）。

当前入院前症状（乏力、黄疸、发热）与既往发作模式一致，提示复发可能。

良性狭窄常见于结石或炎性狭窄（如继发于胆管炎或十二指肠憩室），而恶性狭窄（如胆管癌）通常进行性加重，对取石治疗反应差。但本例中，既往取石后症状缓解，表明初始病因以结石为主。

支持点：十二指肠憩室（既往诊断）是良性病因的促进因素，可导致乳头功能障碍、胆汁淤积和结石形成，但憩室本身非恶性。

当前临床表现和实验室检查更符合良性梗阻伴感染，而非恶性占位

症状以梗阻性黄疸（皮肤巩膜黄染、尿黄、瘙痒）和急性感染（发热、脓毒症）为主：

实验室：总胆红素显著升高（408.5 μmol/L↑，直接胆红素为主），碱性磷酸酶（ALP 625 IU/L↑）和γ-谷氨酰基转移酶（GGT 312 IU/L↑）升高，符合胆汁淤积性肝病，常见于胆道梗阻。

感染标志物极度升高：降钙素原（PCT 30.12 ng/ml↑）、C反应蛋白（CRP 150.1 mg/L↑）、白介素6（IL-6 249.5 pg/ml↑），血培养大肠埃希菌阳性，提示急性胆管炎。这更常见于良性梗阻（如结石导致胆汁淤积和细菌感染），恶性狭窄虽可继发感染，但感染程度如此严重更倾向结石相关。

肾功能不全（肌酐 325-391 μmol/L↑）和电解质紊乱，可解释为脓毒症和脱水所致，非特异于恶性。

恶性狭窄常伴体重减轻、恶病质或持续疼痛，但本例“体重无明显变化”，无相关描述（NRS评分0分），削弱恶性可能性。

影像学特征更支持结石或炎性狭窄，而非明确恶性占位

核心影像学矛盾点：CT报告“胆总管下段闭塞，待排占位或炎性”和“胆总管末端软组织增厚”，这引起恶性怀疑（如胆管癌）。但结合其他影像，更倾向良性解释：

MRCP（2024年1月23日）显示“胆总管稍增宽，其内见低信号结节，较大0.6cm”。这描述一个填充缺损，典型为结石（良性），而非软组织肿块。结石在MRCP中呈低信号，大小0.6cm符合泥沙样结石或小结石（与既往“胆总管泥沙样结石”诊断一致）。

CT增强（影像学1）提到“十二指肠水平段致密影并周围放射状伪影”，这更可能为结石钙化（良性），而非肿瘤征象。

肝内外胆管扩张：CT报告“明显扩张”，但MRCP称“扩张不著”。这种不一致可能由于引流管（PTCD在位）或炎症波动，但扩张程度较轻更倾向间歇性梗阻（如结石移动），而非固定恶性狭窄。

无典型恶性特征：CT和MRCP未描述明确肿块、淋巴结肿大（如“腹膜后未见明显肿大淋巴结影”）、胰管扩张（胰管“显影良好，未见明显扩张”）或肝脏转移灶。软组织增厚可能为炎性水肿（尤其在急性胆管炎背景下），而非肿瘤浸润。

支持点：影像学报告多次建议“结合临床”，而临床病史以结石为主。

整体病程和治疗响应强化良性模式

反复发作性：症状在取石后改善但复发，符合结石残留或再生（尤其患者有糖尿病，增加结石风险）。恶性狭窄通常进行性恶化，而非波动性。

当前治疗：PTCD引流后，实验室指标部分改善（如总胆红素从408.5降至177.8 μmol/L），提示梗阻可逆，更常见于良性病因。

无高危恶性因素：否认肝炎或肝硬化史（降低原发性肝癌风险），肿瘤标志物（如CA19-9）未提供，无法评估。

恶性线索的评估与反驳

年龄因素：70岁增加恶性风险，但年龄本身不足以覆盖具体病史和影像。

软组织增厚：CT中“胆总管末端软组织增厚”需警惕，但可能由急性炎症（如胆管炎）或纤维化（继发于反复结石）引起，而非原发肿瘤。MRCP未重复此描述，且发现“低信号结节”更支持结石。

梗阻位置：胆总管下段闭塞是恶性常见部位（如胰头癌），但本例有明确结石证据（MRCP结节）和憩室，良性可能性更高。

总体权衡：恶性线索（年龄+软组织增厚）被更强的良性证据（结石史、治疗响应、感染主导、无转移）覆盖。

结论

倾向性判断：胆道狭窄原因更可能为良性，具体以胆总管结石（尤其泥沙样结石）或继发性炎性狭窄为主，可能与十二指肠憩室相关。

置信度：中等偏高（约70-80%），因恶性不能完全排除（尤其需增强CT或活检确认）。推荐进一步检查：如ERCP取石+组织活检，或增强MRI以明确软组织性质。

关键推理总结：

良性模式：反复结石病史 + 取石治疗有效 + MRCP见结石样结节 + 严重感染标志物 → 更符合结石/炎性狭窄。

恶性疑虑较弱：年龄和软组织增厚 → 但无进行性恶化、体重减轻或明确占位证据。

**Analysis Process of Incorrect Case 1:**

"Case1：1. The patient is an elderly male, 70 years old, with a chronic disease course. 2. Past medical history: Denied a history of infectious diseases such as hepatitis, tuberculosis, and malaria. He has had diabetes for more than 6 years, did not regularly monitor blood sugar, and was treated with aspart insulin (dose unknown). He has "sick sinus syndrome" but did not pay attention to it or receive treatment. Denied a history of "hypertension" and other medical histories, denied a history of surgery, trauma, blood transfusion, drug and food allergies. The vaccination history is unknown, and denied a history of poisoning. 3. Present illness history: More than 1 month before admission, the patient had fatigue and loss of appetite without obvious trigger. He was examined in an outside hospital, which showed elevated transaminases and abnormal liver function (details unknown). After relevant examinations, he underwent ERCP + ECT + ENBD + DSA at Baoji People's Hospital on October 16, 2023, and was diagnosed with "common bile duct stones, duodenal diverticulum". After receiving liver - protecting and anti - infectious treatment, he was discharged in improved condition. Later, the above symptoms recurred, accompanied by jaundice of the skin and sclera, yellow urine, the urine color was like strong tea, accompanied by itching of the whole body skin. There was no nausea, vomiting, dizziness, headache, cough, chest tightness, shortness of breath and other symptoms. He visited Baoji People's Hospital and underwent ERCP + EST + basket lithotomy, cleaning + ENBD on December 11, 2023, and December 21, 2023, respectively. The intraoperative diagnosis was "common bile duct sandy stones, duodenal diverticulum syndrome", and the condition improved after symptomatic treatment. Ten days before this admission, the patient had fatigue and loss of appetite without obvious incentives, accompanied by fever (body temperature not measured), jaundice of the skin and sclera, yellow urine, and a feeling of fullness in the liver area. There was no obvious nausea, vomiting, hematemesis, melena, hematochezia and other discomforts. So he visited the emergency department of our hospital. Laboratory tests showed: alanine aminotransferase (ALT) 63IU/L↑, aspartate aminotransferase (AST) 88IU/L↑, alkaline phosphatase (ALP) 625IU/L↑, γ - glutamyltransferase (GGT) 312IU/L↑, albumin/globulin (A/G) 0.8↓, potassium (K) 3.47mmol/L↓, sodium (Na) 133.1mmol/L↓, chlorine (CL) 98.0mmol/L↓, creatinine (CRE) 325μmol/L↑, albumin (ALB) 27.9g/L↓, total bilirubin (TBIL) 408.5μmol/L↑, direct bilirubin (DBIL) 375.1μmol/L↑, indirect bilirubin (IBIL) 33.40μmol/L↑, glucose (GLU) 7.97mmol/L↑, total protein (TP) 62.5g/L↓. Blood routine: red blood cell count (RBC) 3.44X10E12/L↓, hemoglobin (HGB) 90g/L↓, hematocrit (HCT) 0.278↓. Blood culture suggested Escherichia coli infection, and the drug sensitivity results suggested that meropenem was sensitive. MRCP showed: common bile duct stones with slightly dilated lumen; the gallbladder was small in volume and poorly visualized, and ascites was detected. Please combine with clinical and abdominal enhanced scan. Abdominal CT showed: 1. Small nodules in the middle lobe of the right lung, the apical - posterior segment of the left upper lobe, and beside the bilateral oblique fissures. A few cords were seen under the pleura of the lower lobes of both lungs. 2. The lower segment of the common bile duct was occluded, to rule out mass or inflammation. The intra - and extra - hepatic bile ducts were significantly dilated. Please combine with clinical; a dense shadow in the horizontal segment of the duodenum. Please combine with clinical. Bilateral renal cysts, right renal calculi. He came to our hospital for further treatment today, and was admitted to the hospital with "1. Obstructive jaundice; 2. Acute cholangitis; 3. Sepsis; 4. Renal insufficiency; 5." by the outpatient department. Since the onset of the disease, the patient's spirit has been fair, physical strength is normal, appetite is normal, sleep is normal, weight has no significant change, defecation is normal, and urination is normal. Body mass index (BMI): 20.20 kg/m2. NRS score is 0. 4. Physical examination: The skin and sclera of the whole body were jaundiced. A PTCD tube was seen on the left side of the abdomen for drainage, and about 300ml of yellow - brown liquid was drained. The abdomen was flat, without gastrointestinal patterns or peristaltic waves, and no varicose veins on the abdominal wall were seen. There was no obvious tenderness in the whole abdomen, no percussion pain in the hepatic flexure, no rebound pain, no muscle tension, Murphy's sign was negative, no mass was palpable in the whole abdomen, and the liver and spleen were not palpable under the ribs. There was no percussion pain in the liver and kidney areas, and the shifting dullness of the abdomen was negative. The bowel sounds were normal on auscultation. 5. Auxiliary examinations: (January 17, 2024, our hospital) Alkaline phosphatase (ALP) 625IU/L↑, γ - glutamyltransferase (GGT) 312IU/L↑, albumin/globulin (A/G) 0.8↓, potassium (K) 3.47mmol/L↓, sodium (Na) 133.1mmol/L↓, chlorine (CL) 98.0mmol/L↓, creatinine (CRE) 325μmol/L↑, indirect bilirubin (IBIL) 33.40μmol/L↑, alanine aminotransferase (ALT) 63IU/L↑, aspartate aminotransferase (AST) 88IU/L↑, albumin (ALB) 27.9g/L↓, total bilirubin (TBIL) 408.5μmol/L↑, direct bilirubin (DBIL) 375.1μmol/L↑, glucose (GLU) 7.97mmol/L↑, total protein (TP) 62.5g/L↓. Blood routine: red blood cell count (RBC) 3.44X10E12/L↓, hemoglobin (HGB) 90g/L↓, hematocrit (HCT) 0.278↓. Blood culture suggested Escherichia coli infection, and the drug sensitivity results suggested that meropenem was sensitive. (January 17, 2024, our hospital) Abdominal CT suggested: 1. Small nodules in the middle lobe of the right lung, the apical - posterior segment of the left upper lobe, and beside the bilateral oblique fissures. It is recommended for follow - up. A few cords were seen under the pleura of the lower lobes of both lungs. 2. The lower segment of the common bile duct was occluded, to rule out mass or inflammation. The intra - and extra - hepatic bile ducts were significantly dilated. Please combine with clinical; a dense shadow in the horizontal segment of the duodenum. Please combine with clinical. Bilateral renal cysts, right renal calculi. (January 23, 2024, our hospital) MRCP suggested: common bile duct stones with slightly dilated lumen, the gallbladder was small in volume and poorly visualized, and ascites was detected. Please combine with clinical and abdominal enhanced scan.

Imaging examination results 1:

CT biliary tract enhancement + 3D imaging, 3D dedicated

Bilateral pleural effusions, mild atelectasis of the lower lobes of both lungs. A solid small nodular shadow is seen in the middle lobe of the right lung. Soft tissue thickening at the end of the common bile duct, a drainage tube can be seen in the intrahepatic bile duct, mild dilation of the intra- and extrahepatic bile ducts, rough wall of the extrahepatic bile duct, a nodular dense shadow and surrounding radial artifacts are seen in the horizontal segment of the duodenum. The liver is normal in shape and size, and a low-density non-enhanced shadow is seen in the left lobe of the liver. The gallbladder is not enlarged, the wall is not significantly thickened, and no abnormal density shadow is seen in the cavity. The spleen is normal in shape and size, the parenchymal density is uniform, and no obvious abnormal enhanced lesions are seen after enhancement. The pancreas is normal in shape and size, the parenchymal density is uniform, and the peripancreatic fat space is clear. No obvious enlarged lymph nodes are seen in the retroperitoneum. There is a small amount of ascites in the abdominal cavity. Small cystic non-enhanced low-density shadows are seen in both kidneys, and a small nodular high-density shadow with a diameter of about 0.4 cm is seen in the right kidney. Mild hydronephrosis of the right kidney and the upper segment of the ureter.

Imaging examination results 2:

MR cholangiopancreatography (MRCP) (1.5T)

MRCP: The intra- and extrahepatic bile ducts are not significantly dilated, the common bile duct is slightly widened, a low-signal nodule is seen inside, the largest is 0.6 cm. The gallbladder is small in volume and poorly visualized, the pancreatic duct is well visualized and no obvious dilation is seen. Ascites is detected.

Abnormal laboratory tests:

White blood cell count #: 9.9 X10E9/L (reference value: 3.50 - 9.50)

Neutrophil percentage: 0.797 (reference value: 0.400 - 0.750)

Lymphocyte percentage: 0.119 (reference value: 0.200 - 0.500)

Absolute neutrophil count: 7.89 X10E9/L (reference value: 1.80 - 6.30)

Red blood cell count #: 3.2 X10E12/L (reference value: 4.30 - 5.80)

Hemoglobin #: 86 g/L (reference value: 130 - 175)

Hematocrit #: 0.269 (reference value: 0.400 - 0.500)

Mean corpuscular hemoglobin: 26.9 pg (reference value: 27.0 - 34.0)

Mean corpuscular hemoglobin concentration: 320 g/L (reference value: 326 - 354)

Total protein*: 57.2 g/L (reference value: 65.0 - 85.0)

Albumin*: 28.5 g/L (reference value: 40.0 - 55.0)

Total bilirubin*: 177.8 μmol/L (reference value: 3.4 - 20.5)

Direct bilirubin: 148.2 μmol/L (reference value: 0.0 - 6.8)

Indirect bilirubin: 29.6 μmol/L (reference value: 6.8 - 12.0)

Alkaline phosphatase: 220 IU/L (reference value: 45 - 125)

γ-glutamyltransferase: 135 IU/L (reference value: 10 - 60)

Albumin/Globulin: 1 (reference value: 1.2 - 2.4)

Leucine aminopeptidase: 78 IU/L (reference value: 30 - 70)

Total bile acid: 41.13 μmol/L (reference value: 0.00 - 15.00)

Triglyceride*: 5.74 mmol/L (reference value: <1.70)

High-density lipoprotein cholesterol*: 0.62 mmol/L (reference value: 0.91 - 2.06)

Urea*: 34.66 mmol/L (reference value: 3.60 - 9.50)

Creatinine*: 391 μmol/L (reference value: 52 - 101)

Uric acid: 466 μmol/L (reference value: 150 - 430)

Cystatin C: 1.81 mg/L (reference value: 0.51 - 1.09)

Carbon dioxide: 13.4 mmol/L (reference value: 20.0 - 29.0)

Troponin I: 0.277 ng/ml (reference value: 0 - 0.03)

Brain natriuretic peptide test (BNP): 720.9 pg/ml (reference value: <38.00)

Heart-type fatty acid binding protein: 30.34 ng/ml (reference value: <6.20)

Prothrombin time: 16 s (reference value: 7.7 - 10.5)

Thrombin time: 20.1 s (reference value: 14.50 - 19.20)

D - dimer: 4.2 mg/L FEU (reference value: <0.50)

Fibrinogen degradation products (plasma): 8.21 μg/mL (reference value: <5.00)

Prothrombin activity: 37.7 % (reference value: 71.00 - 148.00)

PT international normalized ratio: 1.74 (reference value: 0.91 - 1.15)

C - reactive protein: 150.1 mg/L (reference value: <10.0)

Interleukin 6 determination: 249.5 pg/ml (reference value: <7.0)

Procalcitonin quantitative detection: 30.12 ng/ml (reference value: <0.05)

**Analysis Process 1:**

Tendency judgment: The cause of biliary stenosis is more likely to be benign (such as common bile duct stones or inflammatory stenosis)

Reasoning logical chain

Based on the provided medical records, the following is a step - by - step analysis of the logical chain that the cause of biliary stenosis is more inclined to be benign (rather than malignant). The core of the reasoning lies in that the patient has a clear history of cholelithiasis, treatment response, imaging features and infection background, which are more in line with benign causes (such as recurrence of stones or inflammatory stenosis). Although there are some malignant clues (such as age, soft tissue thickening), they are weak and covered by benign factors. The logical chain is divided into key steps, and medical record evidence is cited for support.

1. Starting point: The patient's medical history and previous treatment response strongly support a benign cause

o The patient is a 70 - year - old male (age is a risk factor for malignancy, but not decisive). The course of the disease is recurrent, but the treatment for stones has been effective for many times:

 In October and December 2023, the patient underwent ERCP surgery twice due to similar symptoms (jaundice, abnormal liver function), and was diagnosed with "common bile duct stones" or "common bile duct sandy stones" each time, and the stones were removed. After treatment, the symptoms improved (medical record: "Improved and discharged after liver - protecting and anti - infection treatment").

 The current symptoms before admission (fatigue, jaundice, fever) are consistent with the previous attack pattern, suggesting the possibility of recurrence.

o Benign stenosis is commonly seen in stones or inflammatory stenosis (such as secondary to cholangitis or duodenal diverticulum), while malignant stenosis (such as cholangiocarcinoma) usually progresses progressively and responds poorly to stone - removal treatment. However, in this case, the symptoms were relieved after stone - removal, indicating that the initial cause was mainly stones.

o Supporting point: Duodenal diverticulum (previously diagnosed) is a promoting factor for a benign cause. It can lead to papillary dysfunction, cholestasis and stone formation, but the diverticulum itself is not malignant.

2. The current clinical manifestations and laboratory tests are more in line with benign obstruction with infection, rather than malignant occupation

o The symptoms are mainly obstructive jaundice (yellowing of the skin and sclera, yellow urine, itching) and acute infection (fever, sepsis):

 Laboratory tests: Total bilirubin is significantly increased (408.5 μmol/L↑, mainly direct bilirubin), alkaline phosphatase (ALP 625 IU/L↑) and γ - glutamyltransferase (GGT 312 IU/L↑) are increased, which is in line with cholestatic liver disease, commonly seen in biliary obstruction.

 Infection markers are extremely elevated: Procalcitonin (PCT 30.12 ng/ml↑), C - reactive protein (CRP 150.1 mg/L↑), interleukin 6 (IL - 6 249.5 pg/ml↑), and the blood culture is positive for Escherichia coli, suggesting acute cholangitis. This is more commonly seen in benign obstruction (such as stones causing cholestasis and bacterial infection). Although malignant stenosis can be complicated by infection, such a severe degree of infection is more likely to be related to stones.

 Renal insufficiency (creatinine 325 - 391 μmol/L↑) and electrolyte disorders can be explained by sepsis and dehydration, and are not specific to malignancy.

o Malignant stenosis is often accompanied by weight loss, cachexia or persistent pain, but in this case, "there is no significant change in weight", and there is no relevant description (NRS score is 0), reducing the possibility of malignancy.

3. Imaging features support stones or inflammatory stenosis rather than definite malignant occupation

o Core imaging contradiction: The CT report shows "occlusion of the lower segment of the common bile duct, suspected occupation or inflammation" and "soft tissue thickening at the end of the common bile duct", which raises suspicion of malignancy (such as cholangiocarcinoma). However, combined with other images, a benign explanation is more likely:

 MRCP (January 23, 2024) shows "the common bile duct is slightly widened, and a low - signal nodule is seen inside, the largest is 0.6 cm". This describes a filling defect, which is typically a stone (benign), rather than a soft - tissue mass. Stones show low - signal on MRCP, and a size of 0.6 cm is in line with sandy stones or small stones (consistent with the previous diagnosis of "common bile duct sandy stones").

 CT enhancement (Imaging 1) mentions "dense shadow in the horizontal segment of the duodenum and surrounding radial artifacts", which is more likely to be stone calcification (benign), rather than a tumor sign.

 Dilation of the intra - and extrahepatic bile ducts: The CT report shows "obvious dilation", but MRCP says "not significantly dilated". This inconsistency may be due to the drainage tube (PTCD in place) or inflammatory fluctuations, but the mild degree of dilation is more likely to be intermittent obstruction (such as stone movement), rather than a fixed malignant stenosis.

 No typical malignant features: CT and MRCP do not describe definite masses, enlarged lymph nodes (such as "no obvious enlarged lymph nodes are seen in the retroperitoneum"), pancreatic duct dilation (the pancreatic duct is "well visualized and no obvious dilation is seen") or liver metastases. The soft - tissue thickening may be inflammatory edema (especially in the context of acute cholangitis), rather than tumor infiltration.

o Supporting point: The imaging reports repeatedly suggest "combined with clinical", and the clinical history is mainly related to stones.

4. The overall course of the disease and treatment response strengthen the benign pattern

o Recurrent nature: The symptoms improve after stone - removal but recur, which is in line with the residual or regeneration of stones (especially since the patient has diabetes, increasing the risk of stones). Malignant stenosis usually progresses progressively, rather than being fluctuating.

o Current treatment: After PTCD drainage, some laboratory indicators have improved (such as total bilirubin decreased from 408.5 to 177.8 μmol/L), suggesting that the obstruction is reversible, which is more common in benign causes.

o No high - risk malignant factors: Denied a history of hepatitis or liver cirrhosis (reducing the risk of primary liver cancer), and tumor markers (such as CA19 - 9) are not provided, so it cannot be evaluated.

5. Evaluation and refutation of malignant clues

o Age factor: 70 years old increases the risk of malignancy, but age alone is not enough to override the specific medical history and images.

o Soft - tissue thickening: "Soft - tissue thickening at the end of the common bile duct" in CT needs to be vigilant, but it may be caused by acute inflammation (such as cholangitis) or fibrosis (secondary to repeated stones), rather than a primary tumor. MRCP does not repeat this description and finds a "low - signal nodule" that is more supportive of stones.

o Obstruction location: Occlusion of the lower segment of the common bile duct is a common site for malignancy (such as pancreatic head cancer), but in this case, there is clear evidence of stones (MRCP nodule) and diverticulum, so the possibility of benignity is higher.

o Overall balance: Malignant clues (age + soft - tissue thickening) are covered by stronger benign evidence (history of stones, treatment response, infection - dominated, no metastasis).

Conclusion

• Tendency judgment: The cause of biliary stenosis is more likely to be benign, specifically mainly common bile duct stones (especially sandy stones) or secondary inflammatory stenosis, which may be related to duodenal diverticulum.

• Confidence level: Moderately high (about 70 - 80%), because malignancy cannot be completely excluded (especially enhanced CT or biopsy is needed for confirmation). Recommended further examinations: such as ERCP stone - removal + tissue biopsy, or enhanced MRI to clarify the nature of the soft tissue.

• Key reasoning summary:

Benign pattern: Repeated history of stones + Effective treatment of stone - removal + Stone - like nodule seen on MRCP + Severe infection markers → More in line with stone/inflammatory stenosis.

Weak malignant doubts: Age and soft - tissue thickening → But no progressive deterioration, weight loss or definite evidence of occupation.

**Analysis Process of Correct Case 2:**

" Case2：1.患者中年男性，54岁，慢性病程。2.既往史：发现血压升高4年余，最高压160/90mmHg，口服“硝苯地平缓释片 10mg 1/早、氯沙坦钾片 25mg 1/早”否认“糖尿病”、“心脏病”等病史，4年余前因“胆囊结石”行腹腔镜下胆囊切除术，否认外伤史，否认输血史，否认药物、食物过敏史，预防接种史不详，否认中毒史。 3.现病史：1月余前，患者无明显诱因出现间断腹痛，以上腹部为主，呈胀痛，伴肩背部胀痛不适，无发热，无恶心、呕吐，无明显皮肤、巩膜黄染及尿黄症状，于当地诊所，给予对症治疗（具体不详）后无明显好转，遂就诊于榆林市第一人民医院，检验血常规提示：血红蛋白131g/l；肝功：ALT453.02U/L，AST166.65U/L，总胆红素181.73umol/l，直接胆红素151.55umol/l，间接胆红素30.18umol/l，ALP 546.32U/L。完善胸腹部CT示：右肺左叶磨玻璃结节，建议抗炎治疗后复查；2.右肺上叶钙化灶；3.胰腺颈部异常所见，考虑胰腺癌，邻近的肠系膜上静脉、胆总管下段受侵，其以上平面胆总管、肝内外胆管扩张，主胰管扩张；病变周围脂肪间隙浑浊，考虑受侵；4.副脾、脾大；5。胆囊切除术后。建议上级医院进一步诊治，遂于2023年12月26日来我院就诊，完善腹部CT示：1.考虑胰腺颈部占位，胰腺癌可能性大，胰腺体尾部萎缩，胰管扩张，肝内外胆管扩张。2.肝内多发稍低密度影，转移待排。3.胆囊未见，请结合临床。脾脏体积较大，副脾。4.腹膜后小淋巴结。2023年12月27日行超声引导下经皮胆道穿刺BARD管置入术，现门诊以""胰腺占位（拟行FNA;胆道梗阻PTCD术后;梗阻性黄疸;胆囊切除术后;""收入院。自发病以来，患者精神尚可，体力正常，食欲正常，睡眠正常，体重无明显变化，大便正常，排尿正常。体重指数（BMI）:26.9 kg/m2。NRS评分 1分。4.查体：腹平坦，腹部可见陈旧性手术瘢痕，左侧腹部可见腹腔引流管引流，未见胃肠形及蠕动波，未见腹壁静脉曲张。全腹无明显压痛，无反跳痛，无肌紧张，Murphy征阴性，全腹未扪及包块，肝、脾肋下未及。肝、肾区无叩击痛，腹部移动性浊音阴性。听诊肠鸣音正常。5.辅助检查：（2023年12月21日 榆林市第一人民医院）血常规提示：血红蛋白131g/l；肝功：ALT453.02U/L，AST166.65U/L，总胆红素181.73umol/l，直接胆红素151.55umol/l，间接胆红素30.18umol/l，ALP 546.32U/L。（2023年12月21日 榆林市第一人民医院）胸腹部CT提示：右肺左叶磨玻璃结节，建议抗炎治疗后复查。2.右肺上叶钙化灶；3.胰腺颈部异常所见，考虑胰腺癌，邻近的肠系膜上静脉、胆总管下段受侵，以上平面胆总管、肝内外胆管扩张，主胰管扩张，病变周围脂肪间隙浑浊，考虑受侵；建议胰腺MRI平扫+增强进一步检查；4.副脾、脾大；5。胆囊切除术后。（2023年12月26日 本院）腹部CT提示：1.考虑胰腺颈部占位，胰腺癌可能性大，胰腺体尾部萎缩，胰管扩张，肝内外胆管扩张。请结合MR检查。2.肝内多发稍低密度影，转移待排。3.胆囊未见，请结合临床。脾脏体积较大，副脾。4.腹膜后小淋巴结。

影像学检查结果1：

CT 增强专用,肝胆胰腺增强

胰腺颈部见团状低强化影，边界不清，范围约3.2cm×2.9cm，胰腺体尾部萎缩，胰管可见扩张，肝内外胆管可见扩张。肝脏形态、大小正常，肝实质内见多发类圆形稍低密度影，大者位于肝右后叶下段，大小约1.3cm×1.3cm，增强扫描边缘似轻度强化。胆囊未见，脾脏体积较大，实质密度均匀，增强后未见明显异常强化灶，脾门部见结节影，与脾脏强化一致。胰腺形态、大小未见异常，实质密度均匀，胰周脂肪间隙清晰。双肾形态、大小及密度未见明显异常，双肾周间隙清晰。双侧肾上腺形态、大小及密度未见明显异常。腹膜后见小淋巴结影。腹腔未见积液。

影像学检查结果2：

MR 胰胆管水成像(MRCP)(3.0T)

MRCP：肝内胆管走行正常，未见扩张，胆总管下段局部未见明确显示，其上胆总管见管状低信号影，左右肝管显影良好，管径无增粗，胆囊未见显示。所示胰颈部见斑片状稍长T2信号影，约4.5X4.0cm，远端胰管稍扩张，肝多发类圆形稍长T2信号影，大者约1.0cm。脾大。

异常实验室检查：

异常糖链糖蛋白(TAP)测定：210.06 μm2（参考值：<121.00）

血管内皮生长因子：451.84 pg/ml（参考值：0-160）

白介素1β：19.53 pg/mL（参考值：<12.4）

白介素8：32.85 pg/mL（参考值：<20.6）

干扰素γ：28.84 pg/mL（参考值：<23.1）

癌胚抗原：12.5 ng/ml（参考值：<5.000）

糖链抗原CA19-9：5474 U/ml（参考值：<30.000）

糖链抗原CA125：31.7 U/ml（参考值：<24.000）

凝血酶原时间：11.8 s（参考值：7.7-10.5）

凝血酶原活动度：58.7 %（参考值：71.00-148.00）

PT国际标准化比值：1.29 （参考值：0.91-1.15）

丙氨酸氨基转移酶(ALT)*：154 IU/L（参考值：9-50）

天门冬氨酸转氨酶(AST)*：71 IU/L（参考值：15-40）

白蛋白*：37.2 g/L（参考值：40.0-55.0）

总胆红素*：193.4 μmol/L（参考值：3.4-20.5）

直接胆红素：159.1 μmol/L（参考值：0.0-6.8）

间接胆红素：34.3 μmol/L（参考值：6.8-12.0）

碱性磷酸酶：444 IU/L（参考值：45-125）

γ-谷氨酰基转移酶：528 IU/L（参考值：10-60）

白蛋白/球蛋白：1.1 （参考值：1.2-2.4）

亮氨酸氨基肽酶：157 IU/L（参考值：30-70）

甘油三脂*：4.25 mmol/L（参考值：<1.70）

高密度脂蛋白胆固醇*：0.49 mmol/L（参考值：0.91-2.06）

尿素*：10.27 mmol/L（参考值：3.10-8.00）

胱抑素C：1.24 mg/L（参考值：0.51-1.09）

氯*：97.4 mmol/L（参考值：99.0-110.0）

淋巴细胞百分率：0.154 （参考值：0.200-0.500）

中性粒细胞绝对值：6.39 X10E9/L（参考值：1.80-6.30）

单核细胞绝对值：0.79 X10E9/L（参考值：0.10-0.60）

红细胞计数#：6.06 X10E12/L（参考值：4.30-5.80）

血红蛋白#：122 g/L（参考值：130-175）

血细胞比容#：0.397 （参考值：0.400-0.500）

平均红细胞体积：65.5 fl（参考值：82.0-100.0）

平均血红蛋白含量：20.1 pg（参考值：27.0-34.0）

平均血红蛋白浓度：307 g/L（参考值：326-354）

红细胞分布宽度CV：0.211 （参考值：0.04-0.15）

C反应蛋白：17.9 mg/L（参考值：<10.0）

降钙素原定量检测：0.208 ng/ml（参考值：<0.05）"

**Analysis Process 2:**

倾向性判断：胆道狭窄原因更可能为恶性（胰腺癌导致）

推理逻辑链

以下基于提供的病历资料，逐步分析胆道狭窄原因更倾向恶性（而非良性）的逻辑链。推理核心在于：影像学和实验室检查高度一致地指向胰腺恶性肿瘤，该肿瘤直接侵犯胆总管下段导致狭窄，而良性因素（如结石或炎性狭窄）缺乏支持证据。逻辑链分为关键步骤，引用病历证据支持，并最终总结结论。

起点：影像学特征明确提示恶性占位，直接导致胆道狭窄

多次影像学检查一致描述胰腺颈部占位性病变：

榆林市第一人民医院CT（2023年12月21日）：报告“胰腺颈部异常所见，考虑胰腺癌”，并具体指出“邻近的肠系膜上静脉、胆总管下段受侵”，导致“肝内外胆管扩张”和“主胰管扩张”。这符合恶性胆道狭窄的典型模式（如胰头癌压迫胆总管）。

本院CT（2023年12月26日）：进一步确认“胰腺颈部占位，胰腺癌可能性大”，伴“胰管扩张，肝内外胆管扩张”。增强CT细节：胰腺颈部“团状低强化影，边界不清，范围约3.2cm×2.9cm”，强化了恶性肿瘤的特征（边界不清、低强化提示乏血供肿瘤）。

MRCP：显示“胆总管下段局部未见明确显示”，其上方胆总管扩张（“管状低信号影”），同时“胰颈部见斑片状稍长T2信号影，约4.5X4.0cm，远端胰管稍扩张”。这支持胆道狭窄由胰颈部占位引起，而非良性病因（如结石在MRCP中通常为充盈缺损，但此处未描述）。

关键影像学模式：胰管和胆管同时扩张（“double duct sign”）是胰腺癌的特异性表现，良性狭窄（如结石）罕见此征象。此外，肝内多发低密度/信号影（CT描述“转移待排”，MRCP描述“肝多发类圆形稍长T2信号影”）提示转移可能，进一步支持恶性。

反驳良性可能：无结石证据（如CT/MRCP未报告胆总管结石或钙化）；无炎性狭窄特征（如平滑渐进的狭窄或既往手术吻合口狭窄）。患者虽有胆囊切除史（4年前），但当前影像焦点在胰腺占位，而非术后并发症。

实验室检查：肿瘤标志物显著升高，强烈支持恶性病因

肿瘤标志物极度异常：

CA19-9：5474 U/ml（参考值<30），升高超过180倍。CA19-9是胰腺癌的高度特异性标志物，>1000 U/ml时恶性概率>90%（结合影像学，几乎可确诊）。

CEA：12.5 ng/ml（参考值<5），轻度升高，常见于消化道恶性肿瘤。

CA125：31.7 U/ml（参考值<24），升高，可能提示腹膜或卵巢受累（但本例更关联胰腺癌）。

其他标志物：异常糖链糖蛋白(TAP)和血管内皮生长因子升高，也常见于恶性肿瘤微环境。

肝功能异常符合恶性梗阻性黄疸：

总胆红素193.4 μmol/L（以直接胆红素为主），ALP（444 IU/L）和GGT（528 IU/L）显著升高，表明胆汁淤积。这与胆总管下段受侵导致的机械性梗阻一致。

但无急性感染证据：降钙素原（PCT 0.208 ng/ml）正常，CRP（17.9 mg/L）仅轻度升高。良性狭窄（如结石）常伴明显感染（如胆管炎），但本例无发热或脓毒症描述。

整体实验室模式：肿瘤标志物主导，肝酶异常为继发性，更符合恶性狭窄。良性狭窄（如结石）通常以感染或炎症标志物为主，但此处缺如。

临床表现和病程：符合恶性肿瘤进展模式，而非良性过程

症状：间断上腹痛伴肩背部胀痛（NRS 1分），但无黄疸或尿黄（可能因PTCD引流已部分缓解）。体重稳定（“体重无明显变化”），但胰腺癌早期可无恶病质。

病程：慢性但进行性，1月余前起病，当地对症治疗无效。良性狭窄（如结石）常为反复发作性，对取石或抗感染治疗响应好，但本例无类似病史（既往胆囊切除为4年前，无近期胆石症）。

查体无特异性：无黄疸描述（引流后），无腹部压痛或包块，与胰腺癌早期体征相符。

反驳良性模式：良性胆道狭窄（如术后狭窄或结石）多有黄疸、发热或感染史，但本例初诊时无黄疸症状，且感染标志物不高。

其他恶性证据整合：转移和血管侵犯强化诊断

肝内病变：CT和MRCP均报告肝内多发异常影（CT：“肝内多发稍低密度影，转移待排”；MRCP：“肝多发类圆形稍长T2信号影”）。增强CT描述“肝实质内多发类圆形稍低密度影，增强扫描边缘似轻度强化”，这符合转移瘤特征（良性狭窄罕见肝转移）。

血管侵犯和局部浸润：CT明确描述“邻近肠系膜上静脉、胆总管下段受侵”和“病变周围脂肪间隙浑浊，考虑受侵”，这是恶性肿瘤的直接征象。

淋巴结和脾脏：腹膜后小淋巴结和脾大（副脾），虽非特异性，但在胰腺癌背景下可能反映转移或免疫反应。

良性因素的评估与排除

年龄因素：54岁中年男性，年龄低于典型胰腺癌高发群体（>60岁），但胰腺癌在中年人群并非罕见，且影像学和肿瘤标志物压倒性支持恶性。

既往胆囊切除：4年前手术，但无证据表明当前狭窄与手术相关（如吻合口狭窄）。影像学显示狭窄位于胆总管下段（胰腺水平），而非胆囊管残端。

无结石或炎症支持：无实验室或影像学证据提示胆总管结石（MRCP无充盈缺损）或活动性炎症（如自身免疫性胆管炎）。十二指肠憩室等良性因素未提及。

总体权衡：虽有轻微炎症标志物升高（如IL-1β、IL-8），但程度低，且被更强的恶性证据覆盖。

结论

倾向性判断：胆道狭窄原因更可能为恶性，具体由胰腺癌（胰颈部占位） 导致，肿瘤直接侵犯胆总管下段引起狭窄。

置信度：高（>90%），基于影像学“胰腺癌可能性大”的直接描述、CA19-9显著升高及转移征象。

关键推理总结：

恶性模式：影像学显示胰腺占位伴胰胆管扩张 + CA19-9 >5000 U/ml + 肝内转移灶 → 强烈支持胰腺癌导致胆道狭窄。

良性可能性低：无结石或感染证据 + 对症状治疗无响应 → 无法解释影像和实验室表现。

推荐下一步：确认诊断需组织学检查（如FNA，病历中提到“拟行FNA”），并评估分期（如PET-CT）。PTCD引流已缓解黄疸，但病因治疗需肿瘤综合管理（手术、化疗等）。

**Analysis Process of Correct Case 2:**

" Case2：1. The patient is a middle - aged male, 54 years old, with a chronic disease course. 2. Past medical history: The patient has had elevated blood pressure for more than 4 years, with the highest blood pressure of 160/90 mmHg. He has been taking \"Nifedipine Sustained - Release Tablets 10mg once in the morning, Losartan Potassium Tablets 25mg once in the morning\". He denies a history of \"diabetes\", \"heart disease\", etc. More than 4 years ago, he underwent laparoscopic cholecystectomy due to \"gallbladder stones\". He denies a history of trauma, blood transfusion, drug and food allergies. The history of vaccination is unknown, and he denies a history of poisoning. 3. Present illness: More than 1 month ago, the patient had intermittent abdominal pain without obvious诱因, mainly in the upper abdomen, presenting as distending pain, accompanied by distending pain and discomfort in the shoulder and back. There was no fever, nausea, vomiting, obvious yellowing of the skin, sclera, or urine. He was treated symptomatically at a local clinic (details unknown), but there was no obvious improvement. So he went to the First People's Hospital of Yulin. The blood routine test showed that the hemoglobin was 131g/L; liver function tests showed: ALT 453.02U/L, AST 166.65U/L, total bilirubin 181.73umol/L, direct bilirubin 151.55umol/L, indirect bilirubin 30.18umol/L, ALP 546.32U/L. The thoraco - abdominal CT showed: 1. Ground - glass nodule in the left lobe of the right lung. It is recommended to re - examine after anti - inflammatory treatment; 2. Calcification in the upper lobe of the right lung; 3. Abnormal findings in the neck of the pancreas, considered pancreatic cancer. The adjacent superior mesenteric vein and the lower segment of the common bile duct are invaded. The common bile duct above this level, the intra - and extra - hepatic bile ducts are dilated, and the main pancreatic duct is dilated. The fat space around the lesion is turbid, considered invaded; 4. Accessory spleen, splenomegaly; 5. Status after cholecystectomy. It is recommended to seek further diagnosis and treatment at a superior hospital. So he came to our hospital for treatment on December 26, 2023. The abdominal CT showed: 1. Considered a mass in the neck of the pancreas, with a high possibility of pancreatic cancer. The body and tail of the pancreas are atrophied, the pancreatic duct is dilated, and the intra - and extra - hepatic bile ducts are dilated. 2. Multiple slightly low - density shadows in the liver, metastasis to be excluded. 3. The gallbladder is not seen. Please combine with the clinical situation. The spleen is large in volume, with an accessory spleen. 4. Small retroperitoneal lymph nodes. On December 27, 2023, an ultrasound - guided percutaneous biliary puncture and BARD tube placement was performed. Now the patient is admitted to the hospital with the diagnosis of \"pancreatic mass (planned for FNA; after PTCD for biliary obstruction; obstructive jaundice; after cholecystectomy;\". Since the onset of the disease, the patient's spirit has been good, physical strength is normal, appetite is normal, sleep is normal, there is no significant change in weight, bowel movements are normal, and urination is normal. Body Mass Index (BMI): 26.9 kg/m2. NRS score is 1. 4. Physical examination: The abdomen is flat. There are old surgical scars on the abdomen. An abdominal drainage tube is seen on the left side of the abdomen. No gastrointestinal shape or peristaltic waves are seen, and no varicose veins on the abdominal wall are seen. There is no obvious tenderness, rebound tenderness, or muscle tension in the whole abdomen. Murphy sign is negative. No mass is palpable in the whole abdomen. The liver and spleen are not palpable under the ribs. There is no percussion pain in the liver and kidney areas. The shifting dullness of the abdomen is negative. The bowel sounds are normal on auscultation. 5. Auxiliary examinations: (December 21, 2023, the First People's Hospital of Yulin) Blood routine showed: hemoglobin 131g/L; liver function: ALT 453.02U/L, AST 166.65U/L, total bilirubin 181.73umol/L, direct bilirubin 151.55umol/L, indirect bilirubin 30.18umol/L, ALP 546.32U/L. (December 21, 2023, the First People's Hospital of Yulin) Thoraco - abdominal CT showed: 1. Ground - glass nodule in the left lobe of the right lung. It is recommended to re - examine after anti - inflammatory treatment. 2. Calcification in the upper lobe of the right lung; 3. Abnormal findings in the neck of the pancreas, considered pancreatic cancer. The adjacent superior mesenteric vein and the lower segment of the common bile duct are invaded. The common bile duct above this level, the intra - and extra - hepatic bile ducts are dilated, and the main pancreatic duct is dilated. The fat space around the lesion is turbid, considered invaded. It is recommended to perform pancreatic MRI plain scan + enhancement for further examination; 4. Accessory spleen, splenomegaly; 5. Status after cholecystectomy. (December 26, 2023, our hospital) Abdominal CT showed: 1. Considered a mass in the neck of the pancreas, with a high possibility of pancreatic cancer. The body and tail of the pancreas are atrophied, the pancreatic duct is dilated, and the intra - and extra - hepatic bile ducts are dilated. Please combine with MR examination. 2. Multiple slightly low - density shadows in the liver, metastasis to be excluded. 3. The gallbladder is not seen. Please combine with the clinical situation. The spleen is large in volume, with an accessory spleen. 4. Small retroperitoneal lymph nodes.

Imaging examination results 1:

CT enhanced for specific use, liver, gallbladder and pancreas enhancement

A mass with low enhancement is seen in the neck of the pancreas, with unclear boundaries, about 3.2cm×2.9cm in size. The body and tail of the pancreas are atrophied, the pancreatic duct is dilated, and the intra - and extra - hepatic bile ducts are dilated. The liver is normal in shape and size. Multiple round - like slightly low - density shadows are seen in the liver parenchyma. The largest one is located in the lower segment of the right posterior lobe of the liver, about 1.3cm×1.3cm in size. There seems to be mild enhancement at the edge on enhanced scan. The gallbladder is not seen. The spleen is large in volume, with uniform parenchymal density. No obvious abnormal enhanced lesions are seen after enhancement. A nodular shadow is seen at the splenic hilum, with the same enhancement as the spleen. The pancreas is normal in shape, size, and parenchymal density, and the peripancreatic fat space is clear. The two kidneys are normal in shape, size, and density, and the perirenal space is clear. The bilateral adrenal glands are normal in shape, size, and density. Small retroperitoneal lymph nodes are seen. There is no abdominal effusion.

Imaging examination results 2:

MR cholangiopancreatography (MRCP)(3.0T)

MRCP: The intra - hepatic bile ducts are normal in course, without dilation. The lower segment of the common bile duct is not clearly shown. A tubular low - signal shadow is seen in the common bile duct above this level. The left and right hepatic ducts are well - visualized, and the diameter is not thickened. The gallbladder is not shown. A patchy slightly long T2 - signal shadow is seen in the pancreatic neck, about 4.5X4.0cm in size. The distal pancreatic duct is slightly dilated. Multiple round - like slightly long T2 - signal shadows are seen in the liver, with the largest one about 1.0cm in size. Splenomegaly.

Abnormal laboratory tests:

Determination of abnormal glycan - linked glycoprotein (TAP): 210.06 μm2 (reference value: <121.00)

Vascular endothelial growth factor: 451.84 pg/ml (reference value: 0 - 160)

Interleukin - 1β: 19.53 pg/mL (reference value: <12.4)

Interleukin - 8: 32.85 pg/mL (reference value: <20.6)

Interferon - γ: 28.84 pg/mL (reference value: <23.1)

Carcinoembryonic antigen: 12.5 ng/ml (reference value: <5.000)

Carbohydrate antigen CA19 - 9: 5474 U/ml (reference value: <30.000)

Carbohydrate antigen CA125: 31.7 U/ml (reference value: <24.000)

Prothrombin time: 11.8 s (reference value: 7.7 - 10.5)

Prothrombin activity: 58.7 % (reference value: 71.00 - 148.00)

PT international normalized ratio: 1.29 (reference value: 0.91 - 1.15)

Alanine aminotransferase (ALT)*: 154 IU/L (reference value: 9 - 50)

Aspartate aminotransferase (AST)*: 71 IU/L (reference value: 15 - 40)

Albumin*: 37.2 g/L (reference value: 40.0 - 55.0)

Total bilirubin*: 193.4 μmol/L (reference value: 3.4 - 20.5)

Direct bilirubin: 159.1 μmol/L (reference value: 0.0 - 6.8)

Indirect bilirubin: 34.3 μmol/L (reference value: 6.8 - 12.0)

Alkaline phosphatase: 444 IU/L (reference value: 45 - 125)

γ - Glutamyltransferase: 528 IU/L (reference value: 10 - 60)

Albumin/Globulin: 1.1 (reference value: 1.2 - 2.4)

Leucine aminopeptidase: 157 IU/L (reference value: 30 - 70)

Triglyceride*: 4.25 mmol/L (reference value: <1.70)

High - density lipoprotein cholesterol*: 0.49 mmol/L (reference value: 0.91 - 2.06)

Urea*: 10.27 mmol/L (reference value: 3.10 - 8.00)

Cystatin C: 1.24 mg/L (reference value: 0.51 - 1.09)

Chloride*: 97.4 mmol/L (reference value: 99.0 - 110.0)

Lymphocyte percentage: 0.154 (reference value: 0.200 - 0.500)

Neutrophil absolute value: 6.39 X10E9/L (reference value: 1.80 - 6.30)

Monocyte absolute value: 0.79 X10E9/L (reference value: 0.10 - 0.60)

Red blood cell count#: 6.06 X10E12/L (reference value: 4.30 - 5.80)

Hemoglobin#: 122 g/L (reference value: 130 - 175)

Hematocrit#: 0.397 (reference value: 0.400 - 0.500)

Mean corpuscular volume: 65.5 fl (reference value: 82.0 - 100.0)

Mean corpuscular hemoglobin: 20.1 pg (reference value: 27.0 - 34.0)

Mean corpuscular hemoglobin concentration: 307 g/L (reference value: 326 - 354)

Red blood cell distribution width CV: 0.211 (reference value: 0.04 - 0.15)

C - reactive protein: 17.9 mg/L (reference value: <10.0)

Procalcitonin quantitative detection: 0.208 ng/ml (reference value: <0.05)"

**Analysis Process 2:**

Tendency judgment: The cause of biliary stricture is more likely to be malignant (caused by pancreatic cancer)

Inferential Logical Chain

The following is a step - by - step analysis of the logical chain indicating that the cause of biliary stricture is more likely to be malignant (rather than benign) based on the provided medical records. The core of the reasoning lies in that the imaging and laboratory tests highly consistently point to pancreatic malignant tumor, which directly invades the lower segment of the common bile duct, leading to stricture. There is a lack of supporting evidence for benign factors (such as stones or inflammatory stricture). The logical chain is divided into key steps, citing medical record evidence for support, and finally summarizing the conclusion.

1. Starting Point: Imaging Features Clearly Indicate Malignant Mass, Directly Leading to Biliary Stricture

o. Multiple imaging examinations consistently describe a space - occupying lesion in the pancreatic neck:

. CT of the First People's Hospital of Yulin City (December 21, 2023): The report states "abnormal findings in the pancreatic neck, considering pancreatic cancer", and specifically points out that "the adjacent superior mesenteric vein and the lower segment of the common bile duct are invaded", resulting in "dilation of intra - and extra - hepatic bile ducts" and "dilation of the main pancreatic duct". This is in line with the typical pattern of malignant biliary stricture (such as pancreatic head cancer compressing the common bile duct).

. CT of our hospital (December 26, 2023): Further confirms "space - occupying lesion in the pancreatic neck, high possibility of pancreatic cancer", accompanied by "pancreatic duct dilation, intra - and extra - hepatic bile duct dilation". Details of enhanced CT: "a mass - like hypo - enhanced shadow in the pancreatic neck, with an unclear boundary, about 3.2cm×2.9cm in size", which strengthens the characteristics of a malignant tumor (unclear boundary and hypo - enhancement suggest a hypovascular tumor).

. MRCP shows that "the lower segment of the common bile duct is not clearly visible locally", and the common bile duct above it is dilated ("tubular low - signal shadow"), and at the same time, "patchy slightly long T2 - signal shadow is seen in the pancreatic neck, about 4.5X4.0cm, and the distal pancreatic duct is slightly dilated". This supports that the biliary stricture is caused by the space - occupying lesion in the pancreatic neck, rather than a benign cause (for example, stones usually present as filling defects in MRCP, but this is not described here).

o. Key imaging pattern: The simultaneous dilation of the pancreatic duct and the bile duct ("double duct sign") is a specific manifestation of pancreatic cancer, and this sign is rare in benign strictures (such as stones). In addition, multiple low - density/signal shadows in the liver (described as "suspected metastasis" in CT and "multiple round - like slightly long T2 - signal shadows in the liver" in MRCP) suggest the possibility of metastasis, further supporting malignancy.

o. Refuting the possibility of benignity: There is no evidence of stones (such as no report of common bile duct stones or calcification in CT/MRCP); there are no features of inflammatory stricture (such as smooth and progressive stricture or previous surgical anastomotic stricture). Although the patient has a history of cholecystectomy (4 years ago), the current imaging focus is on the pancreatic mass, not postoperative complications.

2. Laboratory Tests: Significantly Elevated Tumor Markers Strongly Support a Malignant Cause

o. Extreme abnormalities in tumor markers:

. CA19 - 9: 5474 U/ml (reference value < 30), elevated more than 180 - fold. CA19 - 9 is a highly specific marker for pancreatic cancer, and when it is > 1000 U/ml, the probability of malignancy is > 90% (combined with imaging, it is almost diagnostic).

. CEA: 12.5 ng/ml (reference value < 5), slightly elevated, commonly seen in digestive tract malignancies.

. CA125: 31.7 U/ml (reference value < 24), elevated, which may suggest peritoneal or ovarian involvement (but in this case, it is more related to pancreatic cancer).

. Other markers: Elevation of abnormal glycan glycoprotein (TAP) and vascular endothelial growth factor is also common in the micro - environment of malignant tumors.

o. Abnormal liver function is consistent with malignant obstructive jaundice:

. Total bilirubin is 193.4 μmol/L (mainly direct bilirubin), and ALP (444 IU/L) and GGT (528 IU/L) are significantly elevated, indicating cholestasis. This is consistent with mechanical obstruction caused by the invasion of the lower segment of the common bile duct.

. However, there is no evidence of acute infection: Procalcitonin (PCT 0.208 ng/ml) is normal, and CRP (17.9 mg/L) is only slightly elevated. Benign strictures (such as stones) are often accompanied by obvious infection (such as cholangitis), but there is no description of fever or sepsis in this case.

o. Overall laboratory pattern: Tumor markers are dominant, and abnormal liver enzymes are secondary, which is more in line with malignant stricture. In benign strictures (such as stones), infection or inflammatory markers are usually dominant, but they are absent here.

3. Clinical Manifestations and Course of Disease: Consistent with the Progression Pattern of Malignant Tumors, Not a Benign Process

o. Symptoms: Intermittent upper abdominal pain accompanied by shoulder and back distending pain (NRS 1 point), but no jaundice or yellow urine (possibly due to partial relief by PTCD drainage). The weight is stable ("no significant change in weight"), but pancreatic cancer may not present cachexia in the early stage.

o. Course of disease: Chronic but progressive, starting more than 1 month ago, and local symptomatic treatment was ineffective. Benign strictures (such as stones) are often recurrent and respond well to lithotripsy or anti - infection treatment, but this case has no similar medical history (the previous cholecystectomy was 4 years ago, and there is no recent cholelithiasis).

o. Physical examination shows no specificity: No description of jaundice (after drainage), no abdominal tenderness or mass, which is consistent with the early signs of pancreatic cancer.

o. Refuting the benign pattern: Benign biliary strictures (such as postoperative strictures or stones) often have a history of jaundice, fever, or infection, but this case had no jaundice symptoms at the initial diagnosis, and the infection markers were not high.

4. Integration of Other Malignant Evidence: Metastasis and Vascular Invasion Strengthen the Diagnosis

o. Hepatic lesions: Both CT and MRCP report multiple abnormal shadows in the liver (CT: "multiple slightly low - density shadows in the liver, suspected metastasis"; MRCP: "multiple round - like slightly long T2 - signal shadows in the liver"). Enhanced CT describes "multiple round - like slightly low - density shadows in the liver parenchyma, with mild enhancement at the edge during enhanced scanning", which is in line with the characteristics of metastatic tumors (liver metastasis is rare in benign strictures).

o. Vascular invasion and local infiltration: CT clearly describes "invasion of the adjacent superior mesenteric vein and the lower segment of the common bile duct" and "turbid fat space around the lesion, considered invaded", which are direct signs of malignant tumors.

o. Lymph nodes and spleen: Small retroperitoneal lymph nodes and splenomegaly (accessory spleen), although not specific, may reflect metastasis or immune response in the context of pancreatic cancer.

5. Evaluation and Exclusion of Benign Factors

o. Age factor: A 54 - year - old middle - aged male, younger than the typical high - incidence group of pancreatic cancer (> 60 years old), but pancreatic cancer is not rare in middle - aged people, and the imaging and tumor markers overwhelmingly support malignancy.

o. Previous cholecystectomy: The operation was 4 years ago, but there is no evidence that the current stricture is related to the operation (such as anastomotic stricture). Imaging shows that the stricture is located in the lower segment of the common bile duct (at the pancreatic level), not at the cystic duct stump.

o. Lack of support for stones or inflammation: There is no laboratory or imaging evidence suggesting common bile duct stones (no filling defect in MRCP) or active inflammation (such as autoimmune cholangitis). Benign factors such as duodenal diverticulum are not mentioned.

o. Overall balance: Although there is a slight elevation of some inflammatory markers (such as IL - 1β, IL - 8), the degree is low, and it is overshadowed by stronger malignant evidence.

Conclusion

• Tendency Judgment: The cause of biliary stenosis is more likely to be malignant, specifically caused by pancreatic cancer (mass in the pancreatic neck), with the tumor directly invading the distal common bile duct, leading to stenosis.

• Confidence Level: High (>90%), based on the direct imaging description of "high likelihood of pancreatic cancer," significantly elevated CA19-9 levels, and signs of metastasis.

• Key Reasoning Summary:

Malignant Pattern: Imaging shows a pancreatic mass with pancreaticobiliary duct dilation + CA19-9 >5000 U/ml + intrahepatic metastases → strongly supports pancreatic cancer as the cause of biliary stenosis.

Low Probability of Benign Cause: No evidence of stones or infection + no response to symptomatic treatment → cannot explain the imaging and laboratory findings.

• Recommended Next Steps: Confirm diagnosis requires histological examination (e.g., FNA, as mentioned in the medical record "FNA planned") and staging evaluation (e.g., PET-CT). PTCD drainage has alleviated jaundice, but definitive treatment requires comprehensive tumor management (surgery, chemotherapy, etc.).
